# omicsNPC: Applying the Non-Parametric Combination Methodology to the Integrative Analysis of Heterogeneous Omics Data

**DOI:** 10.1371/journal.pone.0165545

**Published:** 2016-11-03

**Authors:** Nestoras Karathanasis, Ioannis Tsamardinos, Vincenzo Lagani

**Affiliations:** 1 Institute of Computer Science, Foundation for Research and Technology—Hellas, Heraklion, Greece; 2 Department of Computer Science, University of Crete, Heraklion, Greece; University of Bonn, Bonn-Aachen International Center for IT, GERMANY

## Abstract

**Background:**

The advance of omics technologies has made possible to measure several data modalities on a system of interest. In this work, we illustrate how the Non-Parametric Combination methodology, namely NPC, can be used for simultaneously assessing the association of different molecular quantities with an outcome of interest. We argue that NPC methods have several potential applications in integrating heterogeneous omics technologies, as for example identifying genes whose methylation and transcriptional levels are jointly deregulated, or finding proteins whose abundance shows the same trends of the expression of their encoding genes.

**Results:**

We implemented the NPC methodology within “omicsNPC”, an R function specifically tailored for the characteristics of omics data. We compare omicsNPC against a range of alternative methods on simulated as well as on real data. Comparisons on simulated data point out that omicsNPC produces unbiased / calibrated p-values and performs equally or significantly better than the other methods included in the study; furthermore, the analysis of real data show that omicsNPC (a) exhibits higher statistical power than other methods, (b) it is easily applicable in a number of different scenarios, and (c) its results have improved biological interpretability.

**Conclusions:**

The omicsNPC function competitively behaves in all comparisons conducted in this study. Taking into account that the method (i) requires minimal assumptions, (ii) it can be used on different studies designs and (iii) it captures the dependences among heterogeneous data modalities, omicsNPC provides a flexible and statistically powerful solution for the integrative analysis of different omics data.

## Introduction

Recent developments in various high-throughput technologies have heightened the need for integrative analysis methods. Nowadays, several studies measure heterogeneous data modalities, as for example methylation levels, protein abundance, transcriptomics, etc., on the same or partially overlapping biological samples/subjects. The key idea is to measure several aspects of the same system in order to gain a deeper understanding of the underlying biological mechanisms. In such settings, a common tasks is identifying molecular quantities that are (a) measured by different omics technologies, (b) related to each other (e.g., associated to the same gene), and (c) that are conjointly affected by the factor(s) under study or associated to a relevant outcome, in a statistically significant way. A typical example is the identification of differentially expressed genes that are also characterized by one or more differentially methylated epigenetic markers [1–3]. Other studies investigate factors that simultaneously enhance the expression of a given protein and the abundance of its related metabolites [4,5]. Another scenario (somewhat less common) is the measurement of the same molecular quantities with different technologies, as for example when previously produced microarray gene expression profiles should be co-analyzed with newly produced RNA-seq data [6].

More in general, the presence of multiple omics data allows the identification of “differentially behaving genes”, i.e., genes that are affected by the factors under study in one or more of the transcription, translation or epigenetic levels.

In this work we introduce and evaluate a novel application of a known statistical methodology, the Non-Parametric Combination (NPC) of dependent permutation tests [7], for the integrative analysis of heterogeneous omics data. NPC has been described in several scientific papers and books [7–9], and it has been applied in the fields of industrial production [10], face/expressions analysis [11] and neuroimaging [12]. However, to the best of our knowledge, this methodology has never been applied in molecular biology. NPC provides a theoretically-sound statistical framework for the integrative analysis of heterogeneous omics data measured on correlated samples. NPC assumes a global null-hypothesis of no association between any of the data modalities and an outcome of interested. This global null-hypothesis is first broken down in a set of “partial null hypotheses”, one for each omics dataset. NPC then uses a permutation procedure that preserves correlations among datasets for simultaneously producing a single p-value assessing the global null-hypothesis, as well as a partial p-value for each partial null-hypothesis.

NPC has several advantages that make it especially suitable for being applied on the analysis of multiple, heterogeneous omics data. Particularly, NPC:

▪ allows the integration of data modalities characterized by different encodings, ranges and data distributions
▪ employs minimal assumptions; particularly, it does not assume independence across data modalities, taking in due account correlations among datasets
▪ provides an interpretable metric as final output, a p-value, which reflects the overall evidence of rejecting or not the global null hypothesis
▪ identifies changes that are supported by at least one modality, assigning a lower p-value to findings which are supported by more modalities
▪ provides the user with the flexibility of weighting differently the information from each dataset based on biological knowledge and experience
▪ exhibits higher statistical power than analyzing each data modality in isolation and thus increase the number of true findings.

NPC is a general methodology, and must be tailored on the idiosyncrasies of the specific data at hand. In order to allow the application of NPC on omics data, we realized the R function omicsNPC, freely available in the STATegRa R-Bioconductor package [13]. The omicsNPC function is able to process and co-analyze different types of omics data, and to combine their results following the NPC principles.

We characterize the performances of omicsNPC in comparison with other methods for the integrative analysis of omics information. To this end simulated as well as real data are used. In the simulated data, each data modality is first analyzed independently using an appropriate statistical approach, and then all data modalities are conjointly analyzed using in turn omicsNPC and other, alternative integrative methods. The results show that the p-values provided by omicsNPC are calibrated, meaning that they follow a uniform distribution when no association is present between any data modality and the outcome of interest. Leek and Storey [14] showed that a number of procedures for controlling the false discovery rate strongly control their respective error measure when applied on calibrated p-value. Moreover, omicsNPC exhibits increased statistical power in all simulated scenarios conducted in this study, by retrieving true findings equally well or better than the other methods, especially when correlation structures are introduced into the data.

We further explored NPC/omicsNPC capabilities on three separate, real data applications: (a) the identification of gene/protein pairs that are deregulated in Glioblastoma, (b) pathway enrichment analysis on expression / methylation profiles in Schizophrenia, and (c) the integration of RNA-seq and microarray expression profiles measured on the same Breast Cancer subjects. The results of these different case-studies pointed out that applying omicsNPC provides more biological insights than analyzing each modality in isolation.

### Related work

The simplest approach adopted in the literature for assessing the conjoint deregulation of multiple, linked molecular quantities consists in analysing each data modality separately, using one of the numerous available methods [15,16], and then try to combine the results in an ad-hoc way. Common integration strategies are verifying whether linked molecular quantities are both associated with the outcome of interest (see for example [17,18]), or graphically inspecting whether their expression / abundance follow similar patterns [19]. These methods do not provide quantitative scores for assessing the overall evidence of conjoint differential behaviour, and the lack of an overall evidence makes the user to decide if the observed differences are relevant or not.

Meta-analysis methods are a more theoretically grounded alternative [20]. Meta-analysis has emerged an effective methodology for data integration, showing that combining information of several studies is more powerful than analysing each study/ data-modality separately, and results can be reproducible and robust by following well defined frameworks [21,22]. Regarding the identification of differentially expressed molecular quantities, meta-analysis methods can be grouped in the following four categories: combining p-values, combining ranks, combining effect sizes and directly merging data modalities [23]. Here we further analyse the first two as more relevant to the problem under discussion; the latter two are usually unfeasible for integrating heterogeneous omics data.

Combining p-values approaches has become popular in the integrative analysis of datasets with common properties [23], where the underlying distributions of the data are assumed to be the same. According to this method datasets are analysed independently using the same statistical test and the resulting p-values, partial p-values, are combined using a proper function, usually Fisher or Stouffer method. The resulting statistic is transformed into a p-value, overall p-value. Partial and overall p-values are generated either parametrically, under the assumption that p-values are uniformly distributed or not-parametrically by permutation-based analysis [24]. However, different data types require different statistical tests, characterized by diverse statistical distribution, and the resulting partial p-values could be inappropriate to combine, as discussed in the supplementary material of a recent study [25].

Combining ranks approaches require the genes to be ranked in each study / data modality according to some measure of differential expression. Then a combined rank is computed by taking the product, the mean, or the sum of the partial ranks. The statistical significance of the combined rank is usually assessed through a permutation-based procedure [23]. The initial impression is that combining ranks methods could be applied in the integrative analysis of different omics data. On one hand the underlying assumptions are minimal: permutation-based testing assumes that samples are independent and identically distributed [26]. However, several questions remain unanswered. In which cases these methods provide calibrated p-values? What is the effect of choosing a combination method over the others? Ranking methods return findings which are supported by all modalities, but what if the researcher is interested in retrieving elements that are supported by at least one modality?

Finally, meta-analysis approaches typically do not consider correlations that may be present among the different data modalities [24,26]. In a typical meta-analysis schema, datasets are assumed to be produced by independent studies. Yet, when different data modalities have been measured on samples from the same subjects / patients, correlations among datasets are expected and should be taken into account. Failure to do so may lead to severe inflation of Type I error rates in the final findings [27]. A considerable amount of literature has been published on extending combining p-values algorithms in order to take correlations into account, which is a non-trivial task [27–29]. The problem is even more complex if a single dataset is expected to be more informative than the other and thus a weighted combining function is desired [29].

## Methods

### The Non-Parametric Combination methodology

**Fig 1:**
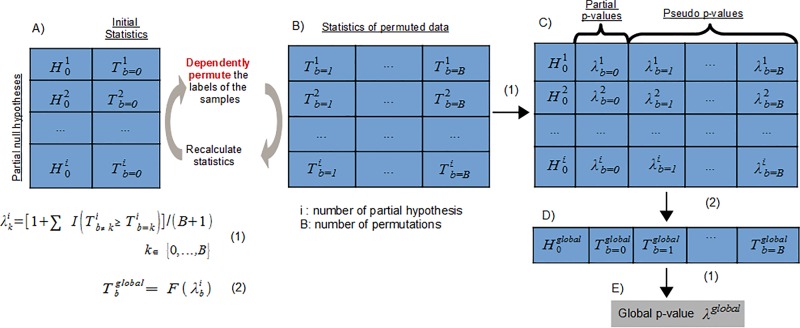
Combining p-values in the NPC framework. See section “Non-Parametric Combination methodology” for an extensive description of the figure.

The omicsNPC function relies heavily on the Non-Parametric Combination (NPC) methodology. Here we use an example to overview the method, state some important points and show the modifications we employ in the case of omicsNPC. Let us assume that we have measured different omics modalities (M^1^, M^2^, …, M^i^) over the same biological samples. We also make the simplistic assumption that each modality provides exactly one measurement for each gene; dropping this assumption is discussed later in the text. Our question of interest is which genes (Gs) are associated with a given outcome of interest / experimental factor, e.g., which genes behave differently between two conditions. NPC is applied by following the steps reported below and depicted in Fig 1.

Step 1: Divide the question of interest to a set of i partial null hypotheses, Fig 1A.

Partial null hypotheses:

▪ $H_{0}^{1}$: the level of G is NOT associated to the outcome of interest in M^1^.

▪ $H_{0}^{2}$: the level of G is NOT associated to the outcome of interest in M^2^.

▪ …

▪ $H_{0}^{i}$: the level of G is NOT associated to the outcome of interest in M^i^.

Global null: all partial null hypotheses are true

Global alternative: at least ONE of partial null is NOT true

Step 2: For each partial null hypothesis i, select a test statistic T^i^ sensitive to the alternative and calculate its value in the observed data, $\left(T_{b=0}^{1}{,\;\text{T}}_{\text{b}=0}^{2},\ldots ,T_{b=0}^{\text{i}}\right)$, Fig 1A. For each partial null hypothesis a suitable statistic should be employed, depending by the nature of the corresponding omics dataset.

Step 3: Estimate the permutation distribution of each test statistic. To this end perform the following B times: a) permute the samples in a manner consistent with the global null hypothesis, and b) calculate the test statistic in the permuted data, $\left(T_{b}^{1}{,\;\text{T}}_{\text{b}}^{2},\ldots ,T_{b}^{\text{i}}\right)$, b ∈ {1,…,B}. Note that each time the samples must be permuted in the same exact way across all data modalities, in order to preserve dependencies among the measurements (Fig 1B). This is an essential technicality of the NPC permutation schema.

Step 4: Calculate the permutation p-values of the partial tests, namely partial p-values, $\left(\lambda_{b=0}^{1}{,\lambda }_{\text{b}=0}^{2},\ldots ,\lambda_{\text{b}=0}^{\text{i}}\right)$. For each statistic T^i^ calculate its partial p-value on the observed data, by comparing its values on the observed data, $T_{b=0}^{\text{i}}$, against its empirical distribution approximated through permutation, $T_{b}^{i}$, b ∈ {1,…,B}. Partial p-values are computed as:

$$\lambda_{b=0}^{i}=\left[1+\Sigma I(T_{b\neq 0}^{i}\geq T_{b=0}^{i})\right]/(B+1)$$ (1)

where the indicator function I( ) assumes value 1 if its argument is true. Compute partial “pseudo” p-value $\left(\lambda_{b}^{1}{,\lambda }_{\text{b}}^{2},\ldots ,\lambda_{\text{b}}^{\text{i}}\right)$, for each permutation b ∈ {1,…,B}, Fig 1C. Note that with respect to the standard estimators, both the numerator and denominator of formula (1) have been augmented by one unit. This is a subtle yet important detail: in a multiple testing context, permutation p-values should never be zero in order to avoid serious inflation of type I error rate, as discussed by Phipson and Smyth [30].

Step 5: Use an appropriate convex function to combine p-values across data modalities into a single “global” test statistic. A final vector of length B + 1 is produced by combining the p-values computed at step 4. The first element $T_{b=0}^{global}$ summarizes the partial p-values $\left(\lambda_{b=0}^{1}{,\lambda }_{\text{b}=0}^{2},\ldots ,\lambda_{\text{b}=0}^{\text{i}}\right)$ observed on the initial datasets, whereas the remaining elements $T_{b}^{global}$, b ∈ {1,…,B} are derived by combining the corresponding “pseudo” p-values, Fig 1D.

Step 6: Calculate a p-value of the global test. Similarly to the partial p-values, the global p-value is computed by equation (1), Fig 1E. Note that also global p-values are never allowed to assume zero value.

Step 7: Assess the evidence of the global null hypothesis by using the global p-value. Each partial hypothesis is evaluated based on its partial p-value. If necessary, both global and partial p-values should be adjusted for multiple testing.

**Fig 2:**
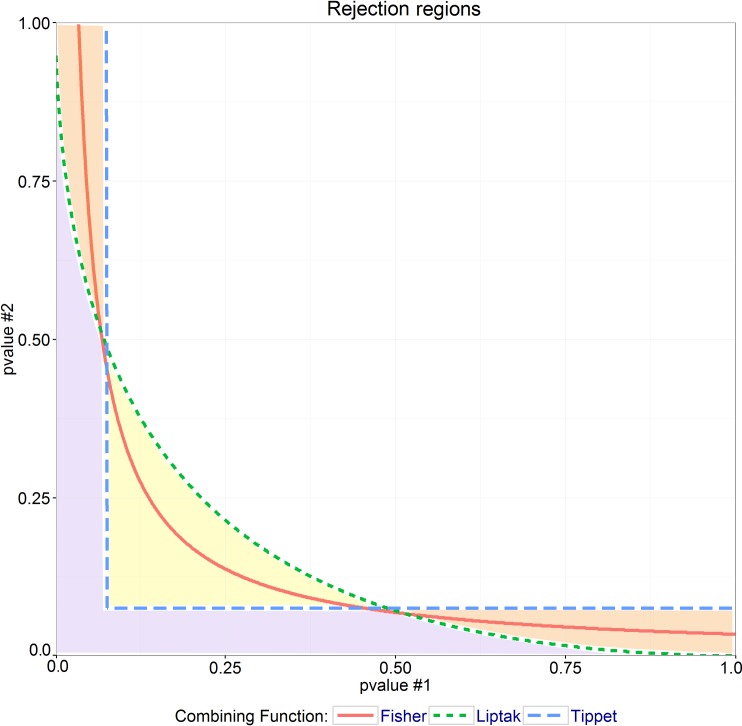
Combining functions’ rejection regions. Rejection regions at significance level 0.15 for the Tippett (blue, dashed), Liptak (green, dotted) and Fisher (red, continuous) functions in combining two independent p-values. The rejection regions are the areas below the respective lines. The Tippett function rejects the global null-hypothesis if at least one of the two partial tests is significant (violet and orange areas); in contrast, the Liptak function can reject the global null hypothesis if both partial tests are not significant, but their combination is (yellow area). However, the presence of a single, extremely high p-value will force the Liptak function to accept the global null hypothesis (orange areas). The Fisher function has an intermediate behavior between the previous two.

An important point of this methodology is the selection of a convex function to combine p-values in step 5. Pesarin and Salmaso [7] discuss three different functions that are suitable for the NPC framework: Tippet, Liptak and Fisher. Each function corresponds to a different rejection region for the global null hypothesis, as shown in Fig 2.

The Tippett function, ${Τ}_{Τ}=\text{max}(1-\lambda_{b}^{i})$, rejects the null hypothesis as soon as any of the partial p-values are below the significance threshold. However, this functions fails in summing up the contribution of several partial p-values that, considered together, may indicate that the global null-hypothesis should be rejected. Thus, the Tippett function yields the most powerful global test when only one or few, but not all, sub-alternatives may occur.

In contrast, the normal combining function, Liptak, $T_{L}=\sum \Phi^{-1}(1-\lambda_{b}^{i})$, would reject the global null-hypothesis when several partial tests are slightly significant, but would accept it if one or more partial tests support it. Fisher's function summarizes p-values as follows, $T_{F}=-2\cdot \sum \text{log}(\lambda_{b}^{i})$, and its behaviour is intermediate between those of Tippett and Liptak. Other functions, such as Mahalanobis and Lancaster's distance could also be used, however we did not employ them in the experiments conducted in this study. Selected combining functions also allow weighting differently the contribution of each data modality. Given a set of positive weights w^i^ such that ∑w^i^ = 1, the Fisher function becomes $T_{F}=-2\cdot \sum w^{i}\cdot \text{log}(\lambda_{b}^{i})$, while the Tippet and Liptak functions become ${Τ}_{Τ}=\text{max}\left(w^{i}\cdot (1-\lambda_{b}^{i})\right)$ and $T_{L}=\sum w^{i}\cdot \Phi^{-1}(1-\lambda_{b}^{i})$, respectively. The weights to use can be selected on the basis of biological or technical considerations (e.g., the expected amount of noise in each data modality).

### Applying the NPC on heterogeneous omics data in practice

#### Mapping measurements across omics modalities

In the simplified explanation presented in the “Non-Parametric Combination methodology” section, each omics dataset was assumed to provide exactly one measurement for each gene. This is often not the case in real studies, where each data modality is usually defined over a distinct set of measurements, and the relation among these sets is often complex and strictly dependent on their biological significance. For example, multiple methylation markers can be associated with the transcriptional activity of the same gene; furthermore, this association is not always certain, especially in case of markers close to the genomic region of the gene but not physically laying on it. The same applies for genomic variants, for the interplay between genes and proteins, or proteins and metabolites.

The NPC methodology requires features measured by different data modalities to be mapped to each other. While the exact mapping strategy can be defined only in the context of each specific study, general guidelines can be provided for the general case where each measurement from data modality M^1^ can be mapped to multiple features in data modality M^2^. A first possible approach is pairing each M^1^ measurement in turn with all their corresponding M^2^ measurements. Another solution is summarizing the information from each group of M^2^ measurements corresponding to a single feature in M^1^. If methylation and expression data are available, the first approach leads to pairing the expression value of each gene with the methylation level of each epigenetic markers attributable to that gene, in turn. In contrast, the second solution requires to compute a single methylation value for each gene.

Integrating more than two modalities requires more complicate mapping strategies. If suitable, a convenient solution is defining a reference set of elements, typically genes, and summarizing all data modalities in such a way that there is one measurement for each element and data type.

#### Analysing partially overlapping samples across datasets

The NPC was originally conceived for co-analyzing datasets measured over the exact same samples. This is often too restrictive in biology, where different datasets might share only part of the samples. For example, proteomics profiles might be available for all the patients involved in a study, while expression profiles are available only for a subset of them. The simplistic solution of co-analyzing only the samples in common clearly discards potentially relevant information. We argue that a better solution is co-analyzing all samples, by preserving the same permutation schema for the overlapping ones. In such a way the correlation among datasets are preserved, while all available information is employed in the analysis.

#### Interpreting global and partial NPC p-values

A significant global p-value λ^global^ indicates that not all data modalities comply with the global null-hypothesis; however, it does not indicate which omics datasets departs from it. To this scope, the partial p-values $\left(\lambda_{b=0}^{1}{,\;\lambda }_{\text{b}=0}^{2},\ldots ,\;\lambda_{\text{b}=0}^{\text{i}}\right)$ can be used for a “post-hoc” analysis. There are a couple of cases of particular interest: (i) the global p-value is significant, along with only one partial p-value. In this case the data modality corresponding to the significant partial p-value drives the results, and should be further investigated; (ii) no partial p-value is significant, even if the global p-value is. This latter case denotes findings that require cumulating information from several omics datasets in order to reach significance, and that would have not being retrieved by analyzing each data modality in isolation.

#### The omicsNPC function

We implemented the “omicsNPC” function within the STATegRa [13] Bioconductor R package for facilitating the application of the NPC methodology on omics datasets. The function accepts an arbitrary number of omics datasets, along with their respective study designs represented as data frames. The datasets are expected to be defined over the same (or overlapping sets of) samples. Datasets defined over disjoint sets of samples can also be analyzed, even though between-datasets correlations cannot be defined in this extreme case, and the NPC methodology becomes equivalent to a permutation-based, non-parametric meta-analysis method. The function internally uses the limma function [15] for computing the association between measurements and the factor under study / outcome of interest, while adjusting for the remaining covariates. The factor under study can be a dichotomous variable (as in case-control studies), a multi-class factor or a continuous outcome. The limma function is directly applied on omics data that can be assumed to be (approximately) normally distributed, as for examples microarray-derived measurements, proteomics, metabolomics, while Next Generation Sequencing (NGS) data (e.g., RNAseq), are pretreated with the voom function [16]. The output of the function is a matrix containing, for each measurement, the partial p-values, as well the global p-values computed with Fisher, Liptak and Tippett functions. A more exhaustive description of the function, as well as a detailed explanation regarding its input arguments and output, can be found in the STATegRa help pages.

### NPC comparative evaluation

We evaluated the performance of the omicsNPC function in an exhaustive comparative study over simulated data. The scope of these analysis is to quantify the capability of each method in retrieving the genes that show a deregulated behavior in one or more heterogeneous datasets.

#### Simulated data

These simulations investigate how omicsNPC and the other methods behave when analysing data characterized by encodings and distributions that commonly occur in biological studies. Particularly, we simulated count data following a negative binomial distribution (representative of RNA-seq data), continuous data distributed (approximately) normally (microarray data), three-level ordinal data (Single Nucleotide Polymorphism, SNP), as well as data generated by the uniform distribution (for reference purposes).

For sake of simplicity, across all experimentations we assume a case-control study design, where the factor under study is a dichotomous variables and no additional covariates are taken into account. The issue of mapping features across different data modalities is not addressed in these analysis, and each data modality is assumed to provide one measurement for each gene.

For each simulation we fixed the total number of measurements, Num_genes, and Num_samples, the total number of samples per group. The RNAseq dataset, namely RNAseq_sim, was simulated using the R package compcodeR [31], while the microarray data, Microarray_sim, were simulated using the R package OCplus [32]. SNP data SNP_sim were simulated by randomly sampling values from the set {0, 1, 2}, while Unif_sim data were sampled from the uniform distribution. For each dataset a number of measurements were deregulated between the two groups, respectively RNAseq_DE for RNAseq data, Microarray_DE for microarray data, SNP_DE for SNP_sim and Unif_DE for Unif_sim. Common_DE indicates the number of elements deregulated at the same time across all modalities, while Cor_level is the correlation among different datasets.

We perform two main experiments using simulated data, named Different Modalities and Correlated Modalities. The first investigates the impact of combining statistics / p-values computed with different statistical tests, while the latter focuses on the impact of correlation among data modalities. In both cases the objective is discriminating deregulated elements from the others.

▪ *Different Modalities*: The purpose of this experiments was to identify how the performances of the different integrative methods are influenced by a) the number of samples, b) the number of modalities and c) the number of permutations. Regarding a), we set the simulation parameters as follows: Num_genes = 2000, RNAseq_DE = 1600, Microarray_DE = 1200, SNP_DE = 800, Unif_DE = 400 and Common_DE = 400. The number of samples per group was varied within {4, 6, 8, 10}. Regarding b), we set the simulation parameters as in a), except for the number of samples that was set to 10 and the number of datasets that was varied between 2 and 4. Regarding c), we generated data for all four data modalities as in a), with the Num_samples set to 10, and the number of permutations varied within {100, 500, 1000, 2000, 5000}. We stress that no correlation structures were added across data modalities in these experiments.
▪ *Correlated Modalities*: In this scenario we simulated two correlated microarray datasets. We set Num_genes = 2000, Num_samples = 10, Microarray_DE = 1000, Common_DE = 1000. We introduced different levels of correlation among the non-differential expressed genes of the two datasets, specifically Cor_level = {0.6, 0.7, 0.8, 0.9, 1}. We forgo introducing a fixed amount of correlation among differentially expressed quantities, since to the best of our knowledge there is no procedure able to ensure both a given amount of correlation and the desired level of differential expression. We also avoid adding within-dataset correlations, since in the NPC framework each gene is analysed across datasets independently by the others, and thus within-dataset correlations would not affect the behaviour of the omicsNPC function.

We analysed RNAseq_sim, Micr_sim, SNP_sim and Unif_sim either (a) independently using respectively voom/limma, limma, the R package scrime [33] and the one sided Wilcoxon rank sum test, or (b) by employing the integrative analysis methods included in the comparative evaluation.

#### Integrative methods included in the comparison

We compared omicsNPC against several integrative analyses methods often used in the literature:

*NPC*^*no-correction*^: we use the standard NPC methodology without correction for zero p-values, in order to evaluate the relevance of this modification.

*RankSum*: this is a standard ranks combining method, where the sum of the ranks is used for providing a statistic of global differential expression.

*omicsNPC*^*RankSum*^: we use the NPC permutation schema for providing p-values for the combined rank computed with the RankSum approach. More specifically, the sum of the ranks is used as global test statistic and its null distribution is generated through permutations, as depicted in panels c) and d) of Figure A in S1 File. The global p-value is calculated by equation (1), see also panel e) of Figure A in S1 File.

*RankProd [26]:* this approach follows the same steps as RankSum but the product of the ranks is used as the global statistic instead of the sum.

*omicsNPC*^*RankProd*^: as in omicsNPC^RankSum^, the omicsNPC permutation schema is employed to generate p-values for assessing the significance of RankProd statistics.

*Combining P-values (CP)*: we use the standard meta-analysis process of combining p-values. Specifically, here we employ the classical Fisher combination method [34]. *Benjamini [35]:* Benjamini and Heller proposed a method for testing whether at least u out of n partial null-hypotheses are false. Their method is valid also when some specific dependency structures hold among the partial p-values, and the global p-value is computed as:

$$\lambda^{Benjamini(u,n)}=\underset{j=1,\ldots ,n-u+1}{\min}\left\{\frac{\left(n-u+1\right)}{j}\lambda_{\left(u-1+j\right)}\right\}$$

where the partial p-values λ_j_ have been ordered so that λ_j_ ≤ λ_j+1_. When u = 1, the null hypotheses of the Benjamini and NPC method coincide. Interestingly, for u = 1 the Benjamini equation reduces to the Benjamini-Hochberg formula for controlling the false discovery rate [36].

#### The partial AUC metric

We evaluated the capability of each method in discriminating differentially expressed quantities by employing the partial AUC, a metric commonly used in Information Retrieval applications. The Receiver Operator Characteristic (ROC) Area Under the Curve (AUC) [37], is a metric which combines sensitivity and specificity information for all possible values of a decision threshold. AUC ranges in the interval of [0, 1], where one corresponds to the perfect rank (i.e., all true findings receive a low p-value), 0.5 corresponds to random ordering and zero to predictions that are perfectly inverted. AUC would evaluate the whole list of p-values, providing a measure of global performance. However, researchers attempting to identify relevant findings often restrict their attention to a few genes, the ones deemed more reliable for subsequent in vitro or in vivo experimental validation, which are usually too expensive or demanding to be performed on all findings. Thus, we are interested in evaluating the partial performances of the methods on the genes corresponding to the lowest p-values. We used a version of AUC known as partial AUC, pAUC [38], which considers a restricted region of the whole sensitivity / specificity curve (specificity in [0.9, 1] for our experimentations). The McClish formula [38] standardizes pAUC values in [0,1], so that the pAUC has the same interpretation of the AUC.

In all experiments conducted in this study, the whole procedure from data simulation to pAUC calculation was performed 20 times and the median pAUC was calculated. Finally, we applied the two sided Wilcoxon test to evaluate the significance of the differences in performances between the best method and all the other ones.

### The joint null criterion

We performed a separated analysis for assessing the correctness (calibration) of the p-values computed by each integrative method through the Joint Null Criterion, JNC [14]. According to the JNC, calibrate methods should provide uniformly distributed p-values when applied on multiple hypothesis testing tasks where all null-hypothesis are true. When the JNC holds, then a number of procedures for multiple testing correction are ensured to strongly control the false discovery rate. We applied the “double Kolmogorov-Smirnov test”, dks, for checking whether the JNC holds, by performing the following process. We used the OCplus package for simulating two uncorrelated or two perfectly correlated microarray datasets, each containing 1000 genes and 20 i.i.d. samples. The samples were randomly and equally subdivided in two groups, and the method to evaluate was applied for assessing the differential expression of each gene. A two sided Kolmogorov-Smirnov test was performed for checking that the resulting p-values follow a uniform distribution. The above process was performed 1000 times, producing 1000 distinct p-values from the Kolmogorov-Smirnov (KS) test. These p-values are also expected to follow a uniform distribution, hypothesis that is assessed using a second Kolmogorov-Smirnov test (hence the name double KS test).

Alternatively, the JNC criterion can also be checked by computing at each iteration the posterior probability that the p-values come from a uniform distribution [14]. Both dks and the posterior probability method are implemented in the “dks” R package [39].

### Applications on real data

We further applied omicsNPC in three different case-studies, for demonstrating (a) omicsNPC applicability on the integration of heterogeneous datasets, and (b) the increase in relevant biological findings obtained by integrating several omics datasets.

#### Integrative analysis of methylation–expression profiles in Schizophrenia

We co-analyzed a gene expression [40] and a methylation dataset [41] measured on whole blood of Schizophrenic patients and healthy controls. Part of the subjects are common between the two datasets, and the data are publicly available in the Gene Expression Omnibus (GEO) repository, GEO id GSE38484 and GSE41037 respectively. First, each dataset was pre-processed independently. The gene expression data were produced with the Illumina HumanHT-12 V3.0 expression beadchip; probesets without annotations and whose coefficient of deviation (defined as the ratio between standard deviation and average value) was below 0.01 were excluded from the analysis. The remaining probesets were collapsed by selecting for each gene the probeset with highest average value. Subjects GSM943305 and GSM943331 were deemed outliers and excluded after visual inspection of the Principal Component Analysis (PCA) plots. Methylation data were measured with the Illumina HumanMethylation27 BeadChip, and were adjusted for batch effects using the ComBat method [42] (known batches were indicated by the original authors in private communications). Methylation probesets lacking annotations or whose methylation site was further than 500 kilo bases from the Transcription Start Site (TSS) of the closest gene were excluded, as well as probesets whose values spanned an interval less than 0.1. Following the single-dataset preprocessing, each methylation site was linked to the closest gene measured in the expression dataset (cpg-to-the-closest-gene mapping, [43]); unmatched genes or methylation sites were excluded. The final expression and methylation datasets contain 200 (105 cases) and 658 (293 cases) subjects, respectively, out of which 50 subjects are in common. The expression dataset is defined over 4962 genes, while 6428 methylation sites are reported in the methylation data.

#### Deregulation of protein and gene expression levels across Glioblastoma subtypes

Proteomics and transcriptomics profiles of Glioblastoma patients were downloaded from the The Cancer Genome Atlas (TCGA) repository. We used the data as preprocessed by the TCGA consortium (“Level 3” preprocessing); expression data were measured with the Affymetrix HT Human Genome U133 microarray, while proteomics information was produced with the reverse phase protein array technique [44]. Both datasets were adjusted for known batches with the ComBat method, and 114 protein measurements were associated to their corresponding genes accordingly to the annotation of the data producer (RPPA Core Facility, MD Anderson Cancer Center, University of Texas). We focuses on the comparison between ‘Classical” and “Mesenchymal” Glioblastoma. At the end of the preprocessing, the transcriptomics and protein datasets contained respectively 315 (159 Mesenchymal) and 98 (45 Mesenchymal) samples, out of which 97 were in common.

#### Co-analyzing NGS and microarray expression data in Breast Invasive Carcinoma

The expression profiles of 16 distinct BReast invasive CArcinoma (BRCA) patients were downloaded from TCGA repository. For each patient two types of tissues are available, i.e., tumor and healthy ones, and each tissue is profiled with three different technologies, “RNAseq”, “RNAseqV2” and “Exp-Gene”, leading to a total of six expression profiles for each patient. RNAseq and RNAseqV2 are both produced by mRNA sequencing but with slightly different pipelines, while the Exp-Gene dataset is generated with microarray technology. The selected 16 patients belong to the same experimental batch, namely batch 93. We used the data as pre-processed by the TCGA consortium at “Level 3”, meaning that count matrices are provided for the sequencing datasets and normalized expression values for the microarray samples. In total, 16146 genes were in common among all datasets and were subsequently analyzed.

In all case-studies the datasets were first analyzed in isolation with limma (voom/limma for RNAseq data). Furthermore, datasets were co-analyzed with omicsNPC by setting the number of permutations to 10000. All Schizophrenia and Glioblastoma analyses were corrected for age and gender.

## Results

### The JNC applied on integrative analysis methods

We applied the joint null criterion to evaluate whether the p-values produced by each method follow the desired uniform distribution when the null-hypotheses are all true. To this end, we simulated 1000 times either two uncorrelated or two perfectly correlated microarray datasets, and we applied the double KS test process.

The omicsNPC function exhibited the same behavior in both the correlated and uncorrelated scenario, generating calibrated p-values with all combining functions (Figures B and G in S1 File). NPC^no-correction^ generated slightly non-calibrated p-values, in both uncorrelated and correlated scenarios. In the uncorrelated scenario (Figure C in S1 File), the dks p-values for the Fisher and Liptak methods were one order of magnitude lower than omicsNPC respective dks p-values (p-value = 0.08 and 0.07, respectively). NPC^no-correction^ combined with the Tippett method was found to generate non-calibrated p-values (dks p-value = 0.012). In the correlated scenario NPC^no-correction^ generated non-calibrated p-values, regardless of the combining function used (Figure H in S1 File). Due to these findings we deemed the correction in the formula for computing permutation-based p-values necessary, and we use only omicsNPC in the subsequent experiments. Interestingly, assessing the JNC is not trivial when the sum or the product of the ranks are used as combining functions in conjunction with the NPC permutation schema. In both uncorrelated and correlated scenarios, omicsNPC^RankSum^ and omicsNPC^RankProduct^ obtain a dks p-value ≈ 0 (Figures D and I in S1 File, panels a and b). However, the empirical distribution function of the first level KS test p-values is clearly shifted toward high p-values, indicating that at each repetition the KS test accepts the null-hypothesis of uniformly distributed p-values, as also suggested by distribution of the posterior probabilities. Thus, assessing the JNC for these two methods seem quite controversial. Notably, to the best of our knowledge, this is the first time that the distribution of NPC p-values is evaluated through the joint null criterion.

We used an approximate algorithm [45] for estimating RankProd p-values, over which we assessed the JNC for this method. The RankProd behaves similarly to omicsNPC^RankSum^ and omicsNPC^Rankroduct^ in the uncorrelated scenario (Figure D in S1 File, panel c). However, in the correlated scenario this method produces uncalibrated p-values, as evident in the Q-Q plot of Figure I in S1 File, panel c (dks p-value ≈ 0). Also the CP method produced calibrated p-values in the non-correlated scenario (dks p-value = 0.8, Figure E in S1 File), while producing uncalibrated p-values when correlation structures were included in the data, dks p-value ≈ 0 (Figure J in S1 File). Finally, the Benjamini method produced calibrated p-values in both uncorrelated and correlated scenarios (Figures F and K in S1 File, respectively).

### Results of simulation studies

**Fig 3:**
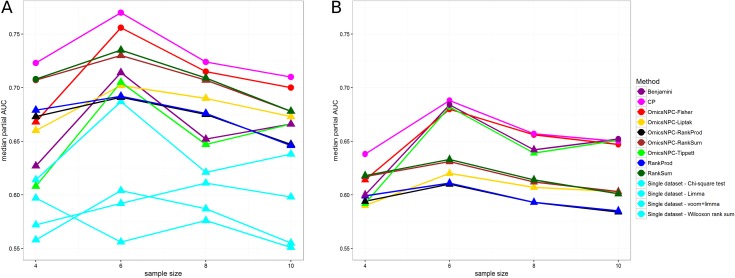
Median pAUCs in relation to the number of samples in the different modalities scenario. Each line corresponds to a specific method. The x axis represents the number of samples per group used in the simulations, and y axis the median pAUCs. Circles suggest that the observed differences from the best method were not statistically significant at level ≤ 0.05. A) Only genes which exhibited the same behaviour across datasets were taken into account. The integrative analysis yielded better results regardless the employed method. CP and omicsNPC^Fisher^ were the best method in most cases. B) All genes were considered in this analysis. Again, omicsNPC^Fisher^ and CP achieved better performance than other methods in most cases; the Benjamini method was also statistically indistinguishable from the best performing method when the sample size was 6 or 10.

We evaluated the capability of each method in retrieving true positive findings on simulated data, where we could assess their performances on the known ground truth. In the different modalities scenario we simulated several types of data (RNAseq_sim, Microarray_sim, SNPs_sim, Unif_sim) and analyzed them either independently or by employing integrative analysis methods. We evaluated performances by employing the pAUC metric. First, we restricted our analysis on genes that have the same behavior across all modalities, i.e., they are either deregulated in all datasets or in none of them, and examined the effect of the sample size on the performances. Overall, integrative approaches performed better in identifying differential expressed genes, in comparison with methods that analyze each data modality independently (Fig 3A, Table A in S1 File).

This stands also for low sample sizes (samples per group = 4). In all cases CP performed equally or slightly better than omicsNPC, however the observed differences were often not statistically significant: when the sample size was equal to 4, CP performed equally well with omicsNPC^RankSum^ and RankSum, whereas for bigger sample sizes CP was as well performing as omicsNPC^Fisher^.

When all genes are taken into account, CP performed better than other methods for moderated sample size, samples = 4, while omicsNPC^Fisher^ performed equally well with CP in all other cases (Fig 3B, Table B in S1 File). Benjamini was also one of the best methods when the number of samples was six and ten. Applying single-dataset methods would have not been meaningful in this case, since each dataset has different sets of deregulated quantities and results would not be comparable.

**Fig 4:**
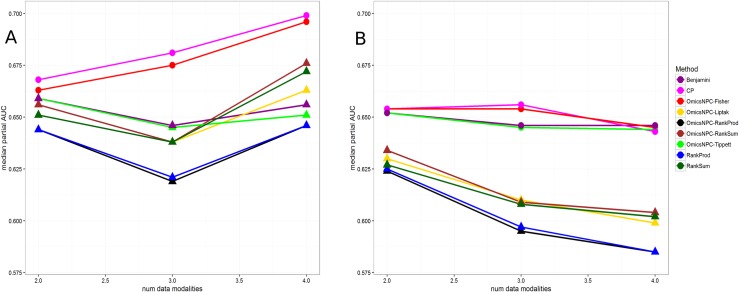
Median pAUCs in relation to the number of modalities analysed in the different modalities scenario. Each line corresponds to a specific method. The x axis represents the number of modalities analysed, and the y axis the median pAUCs. Circles suggest that the observed differences from the best method were not statistically significant at level ≤ 0.05. A) Only genes which exhibited the same behaviour across all datasets were taken into account. In this experiment only the integrative approaches were evaluated. CP and omicsNPC^Fisher^ were the best method in all cases. B) All genes were considered in this analysis. omicsNPC^Tippett^, omicsNPC^Fisher^, CP and Benjamini achieved better performance than other methods.

Furthermore, we evaluated the effect of the number of modalities on integrative methods’ performances. Similarly to above, first we considered only the genes that behaved similarly across all modalities. As evident from Fig 4A and the Table C in S1 File, CP and omicsNPC^Fisher^ performed equally well. Furthermore, adding more modalities increased the performance of omicsNPC^Fisher^, omicsNPC^Liptak^, omicsNPC^RankSum^ and RankSum. On the other hand when we performed the same analysis taking into account all genes (Fig 4B and Table D in S1 File), omicsNPC^Tippett^, omicsNPC^Fisher^, CP and Benjamini performed equally well in all cases. It is worth noting that the performances of all ranking methods and omicsNPC^Liptak^ were slightly decreasing as we were adding more data modalities.

Also, we examined the performance and the computational time of the algorithms in relation with the number of permutations (Figure L and Table E in S1 File). We observed that the number of permutations had a relatively low influence on the performance of the ranking approaches and omicsNPC^Liptak^, while omicsNPC^Tippett^, omicsNPC^Fisher^ and CP reached their maximum performances at 1000 permutations. In addition, the computational time of omicsNPC presented a linear relation with the number of permutations.

**Fig 5:**
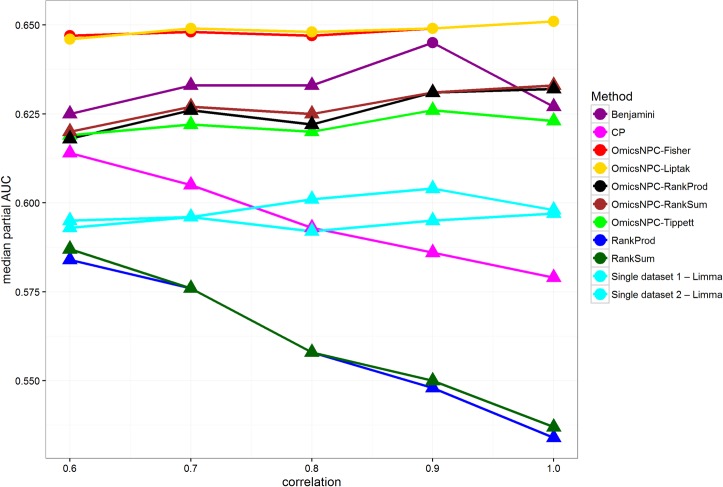
Median pAUCs in relation to correlation structures. Each line corresponds to a specific method. The x axis represents the level of correlation introduced between the datasets, and the y axis the median pAUCs. Circles suggest that the observed differences from the best method were not statistically significant at level 0.05. omicsNPC^Fisher^ and OmicsNPC^Liptak^ were the best methods in all cases. All functions employing omicsNPC framework, as well as Benjamini, showed a stable performance regardless the correlation intensity. On the other hand methods which did not account for the correlations structures, showed decrease in performance as the correlation was higher. Note that single-dataset analyses, sky blue lines, performed better than these methods when strong correlations are present.

In the correlated modalities scenario we introduced different levels of correlation between two microarray datasets. In all cases omicsNPC^Fisher^ and omicsNPC^Liptak^ performed identically, and they were the best performing methods (Fig 5, Table F in S1 File). All combining function that employed the NPC permutation framework, as well as the Benjamini method, showed a stable performance, as they were able to take into account the correlation structures between the datasets. On the contrary, the performance of methods which did not correct for correlations (CP, RankProd, RankSum), behaved inversely as the level of correlation. It is worth noting that after a specific correlation threshold, analyzing datasets in isolation with limma provides better performances than methods overlooking between-datasets correlation structures.

### Results on the real-data case-studies

The results on the three cases-studies underline how the non-parametric combination framework is able to provide additional, biological insights with respect to analyzing each dataset in isolation.

In the breast invasive carcinoma, BRCA, two RNA-seq and one microarray gene expression dataset were generated from the same cancer patients; thus high between-datasets correlation structures should be expected. Indeed, the median Spearman correlation between the two sequencing dataset is 0.93, while measurements in the Exp-Gene have a median Spearman correlation with their respective counterparts in RNAseq and RNAseqV2 of 0.6 and 0.57, respectively. This application seems an ideal test-bed for omicsNPC, and indeed we observe that applying omicsNPC on all data-types allows to retrieve a higher number of differentially expressed genes than analyzing each dataset in isolation, and this effect is independent by the combining function or significance threshold used on the FDR adjusted p-values (Table G in S1 File). For example, at 0.05 FDR level the Liptak combination function provides 7429 findings, versus a maximum of 7116 retrieved analyzing the RNAseq dataset alone. Furthermore, 133 out of the 7429 Liptak significant genes are not significant in single-dataset analyses, and 24 out of these 133 genes were barely significant (adjusted p-value in [0.05–0.1]) for both RNA-seq and the microarray data. This indicates how integrating several data sources with OmicsNPC allows to retrieve findings that would not be identified by analyzing each omics dataset in isolation. Interestingly, an enrichment analysis performed on these 24 genes over the Disease Ontology of the OBO Foundry [46] shows that six out of the ten most enriched diseases are ovarian-related cancers (Table 1), a class of malignancies known to share similar hormonal [47] and genetic bases [48] with breast cancer.

**Table 1:** Disease Ontology enrichment over the 24 BRCA genes identified solely by omicsNPC (Liptak combining function).

ID	Description	p-value	Adjusted p-value
DOID:3369	peripheral primitive neuroectodermal tumor	0.000550441	0.131004856
DOID:3713	ovary adenocarcinoma	0.001650812	0.182356547
DOID:10534	stomach cancer	0.008751988	0.182356547
DOID:1856	Cherubism	0.009691775	0.182356547
DOID:3605	ovarian cystadenocarcinoma	0.009691775	0.182356547
DOID:2151	malignant ovarian surface epithelial-stromal neoplasm	0.009845306	0.182356547
DOID:2152	ovary epithelial cancer	0.009845306	0.182356547
DOID:4001	ovarian carcinoma	0.009845306	0.182356547
DOID:5828	endometrioid ovary carcinoma	0.011298616	0.182356547
DOID:2394	ovarian cancer	0.012482471	0.182356547

The table reports the enrichment result computed with hypergeometric test (function “enrichDO” from the R package “DOSE”) over the 24 genes identified by omicsNPC equipped with the Liptak combining function and deemed barely significant by the single-dataset analyses.

**Fig 6:**
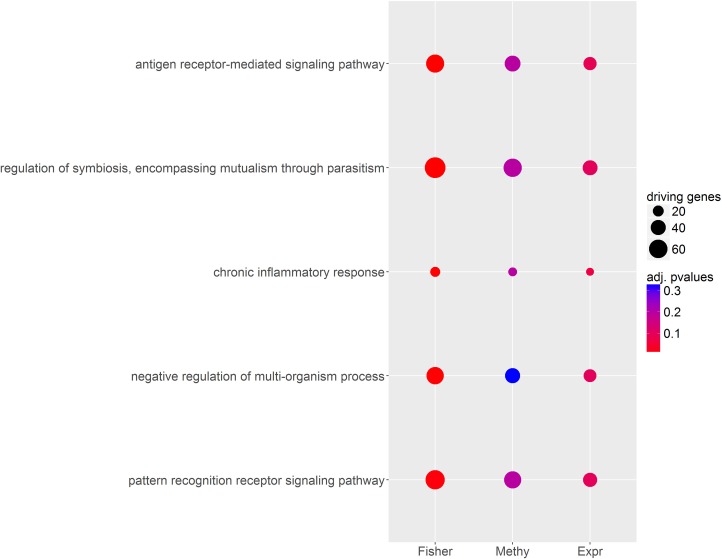
Pathway enrichment analysis in the Schizophrenia case-study. Some pathways are enriched according to omicsNPC (Fisher combining function) but not for the single-dataset analysis (FDR level 0.05 in all cases); the 5 most enriched of such pathways are reported on the y-axis. Each dot represents the significance (color) and number of deregulated genes (size) in the respective pathway according to omicsNPC^Fisher^ (Fisher), methylation (Methy), or transcriptomics data (Expr).

Applying omicsNPC on the Schizophrenia data further confirms NPC’s capabilities of retrieving relevant biological findings. At an FDR level of 0.05, analyzing methylation data alone identifies 3194 genes with at least one altered methylation site, against 1892 genes whose expression is deregulated. Pairing methylation and expression data with omicsNPC and the Fisher combining function leads to the identification of 3844 genes deregulated at the methylation / expression level. We analyzed the enrichment of these significant genes in KEGG [49], Gene Ontology Biological Processes [50] and Reactome [51] pathways using the hypergeometric test, and excluding pathways with less than 20 or more than 200 elements (3918 pathways to test in total). The enrichment analysis identified sixty-eight pathways enriched for omicsNPC findings, out of which thirty-nine were not significant when the enrichment was performed on each data modality in isolation (FDR level 0.05 in all cases, S2 File). Fig 6 reports the five most significant of them, comparing their level of significance according to omicsNPC against methylation and expression data alone. The first pathway, the biological process “antigen receptor-mediated signaling pathway” (GO id GO:0050851), represents a set of molecular signals that are initiated in B or T cells by the cross-linking of an antigen receptor. Interestingly, Schizophrenia has been found associated to antigen processing and Human Leucocyte Antigen (HLA) genes in numerous studies [52–54], findings that corroborate an implication of the immune system in the disease [55,56]. Similarly several studies have hypothesized a role of Chronic Inflammation in Schizophrenia [57–60]. Finally, deficiencies in Toll-like receptor-2, a family of pattern recognition receptors, have been demonstrated to induce Schizophrenia-like behaviors in mice [61].

In the Glioblastoma case-study, most of the proteins show a deregulation at 0.1 FDR level between Mesenchymal and Classical Glioblastoma; 53 deregulated proteins in the proteomics data, 64 differentially expressed genes in the transcriptomics data, and 95 deregulated elements in both data types combined (omicsNPC, Fisher combining function). Also in this case, omicsNPC retrieves additional findings that seem to have biological relevance: proteins Myc, MSH6 and STAT3 were significant at 0.1 FDR level for omicsNPC, even if they were not significant when proteomics and transcriptomics data were analyzed in isolation. Genes encoding Myc, MSH6 and STAT3 are well known for their role as oncogenes [62–64]. More importantly, STAT3 has been found as an initiator and master regulator of brain tumor mesenchymal transformation [65]; MSH6 mutations have reported to affect treatment response in Glioblastoma [66]; and Myc has been found separately associated to glioma/glioblastoma [67][68] and mesenchymal transition of epithelial cells [69].

## Discussion

In this study we have shown that the Non-Parametric Combination, NPC, is a valuable methodology for the integration of heterogeneous omics datasets. NPC is particularly useful for identifying molecular quantities that, considered together, are deregulated / associated to an outcome of interested in a statistically significant way.

We realized the omicsNPC function for facilitating the analysis of omics data within the NPC framework. The function is freely available in the R Bioconductor package STATegRa, and is able to address a variety of different study designs as well as analyzing datasets having only part of their samples in common.

We contrasted omicsNPC against two rank-based and two parametric approaches. The results showed that OmicsNPC^Fisher^ performed better than other methods in most scenarios conducted in this study. When no correlation structures were present in the data, the CP method was slightly more performant than omicsNPC^Fisher^, with the observed differences often being not statistically significant. In contrast, when between-datasets correlation structures were introduced, omicsNPC outperformed all other methods, while approaches that did not take those correlations into account performed poorly, achieving lower performances than single-dataset analyses for moderate to high correlation levels (Fig 5).

Furthermore, we applied the joint null criterion, JNC, on all methods under study employing correlated as well as uncorrelated datasets. The omicsNPC function always presented the same unbiased behavior, when Liptak, Tippett or Fisher combining functions were used (Figures B and G in S1 File), and permutation-based p-values were corrected for avoiding reaching zero. RankProd and CP produced uncalibrated p-values when correlated datasets were considered (Figures I and J in S1 File), while the Benjamini method showed to comply with the JNC in all simulations (Figures F and K in S1 File).

Furthermore, we explored NPC as a general framework for integrative analysis, by using ranking methods as combining functions (Figure A in S1 File). The key idea is using the permutation strategy of the NPC framework for allowing different integration methods, RankSum and RankProd in our analysis, to correctly address between-datasets correlations. Indeed, the performances of the omicsNPC^RankProd^ and omicsNPC^RankSum^ methods were not negatively affected by the level of correlation among data modalities, contrarily to the original RankSum and RankProd approaches (Fig 5). Also the results of the dks tests did not indicated any difference due to the presence of correlation (Figures D and I in S1 File), even though it is not clear whether the JNC holds for omicsNPC^RankProd^ and omicsNPC^RankSum^.

Finally, we applied OmicsNPC on real data from three different case-studies. Taken together, the results on the three real-data case-studies show that omicsNPC is (a) versatile enough to be used for the integration of different omics data; (b) able to include co-variates into the analysis and process sets of datasets that share only part of the samples; (c) finally, omicsNPC have shown, at least in the context of our analysis, to produce additional, relevant biological insights with respect to analyzing data modalities in isolation. Particularly, we concentrated on results deemed statistically significant by omicsNPC but discarded by single-dataset analysis, retrieving interesting findings both at gene (Myc, STAT3 and MSH6 genes in the Mesenchymal vs. Classical Glioblastoma case study), pathway (the antigen and pattern recognition signaling pathways in the Schizophrenia example) and disease levels (gynecologic cancer diseases in BRCA).

In conclusion, several important features of the NPC methodology make it appropriate for the integrative analysis of omics data. First, the underlying assumptions are minimal: NPC permutation schema requires observations to be exchangeable under the null hypothesis, a requirement common in permutation-based approaches. Furthermore, the partial p-values are assumed to be adequate for assessing the partial null-hypothesis, marginally unbiased and consistent [70], while the combining functions must respect a set of mild assumptions [71]. The possibility of equipping NPC methods with different combining functions provides the method with great flexibility: researchers can select the function that best reflects their expectative on the data or that are most suitable for answering their specific scientific questions. Last but not least, NPC frees the researcher from the necessity to define and model the dependence relations among different data modalities and partial tests.

Finally, we would like to underline the limitations of the present study. First, we restricted our simulations to the special case where exactly one measurement per gene is provided by each data modality, and this is rarely the case in real applications. All analyses were performed solely in the context of case-control studies, without taking into consideration other types of study designs. The performances of the different integrative methods, as well as the joint-null criterion, were evaluated only on simulated data, since the ground-truth necessary for the evaluation is not available in the case-studies on real data.

## Conclusions

To the best of our knowledge, this is the first study investigating the applicability of the Non-Parametric Combination methodology in the analysis of heterogeneous omics data. Our results indicate omicsNPC and the NPC framework as versatile and valuable tools for performing integrative analyses in biological studies.

## Supporting Information

S1 File This file contains Figure A, which explain OmicsNPC employing ranking methods, Figures B–K which illustrate the diagnostic plots of the joint null hypothesis criterion. Further it includes Figure L which demonstrates how omicsNPC performances are influenced by the number of permutations. Finally it includes Tables A–F, which report the median pAUCs values for the experimentations on simulated data and their respective significance levels. Table G reports the number of deregulated genes in the BRCA case-study.
(PDF)

S2 File Results of the enrichment analysis on the Schizophrenia case-study. (XLSX)
