# Supplementary material for: omicsNPC: Applying the Non-Parametric Combination Methodology to the Integrative Analysis of Heterogeneous Omics Data
Source: PLoS One. 2016 Nov 3;11(11):e0165545. doi: 10.1371/journal.pone.0165545 (PMC5094732; doi:10.1371/journal.pone.0165545)
Supplement: S1 File — This file contains Figure A, which explain OmicsNPC employing ranking methods, Figures B–K which illustrate the diagnostic plots of the joint null hypothesis criterion. Further it includes Figure L which demonstrates how omicsNPC performances are influenced by the number of permutations. Finally it includes Tables A–F, which report the median pAUCs values for the experimentations on simulated data and their respective significance levels. Table G reports the number of deregulated genes in the BRCA case-study. (PDF) [file pone.0165545.s001.pdf]

# Supplementary material for the manuscript: “omicsNPC: applying the Non-Parametric Combination methodology to the integrative analysis of heterogeneous omics data”

Nestoras Karathanasis[1], Ioannis Tsamardinos[2], Vincenzo Lagani[2*]

[1]Institute of Computer Science, Foundation for Research and Technology - Hellas, Heraklion, Greece

[2]Department of Computer Science, University of Crete, Heraklion, Greece.

[*]Corresponding author

Email: vlagani@csd.uoc.gr

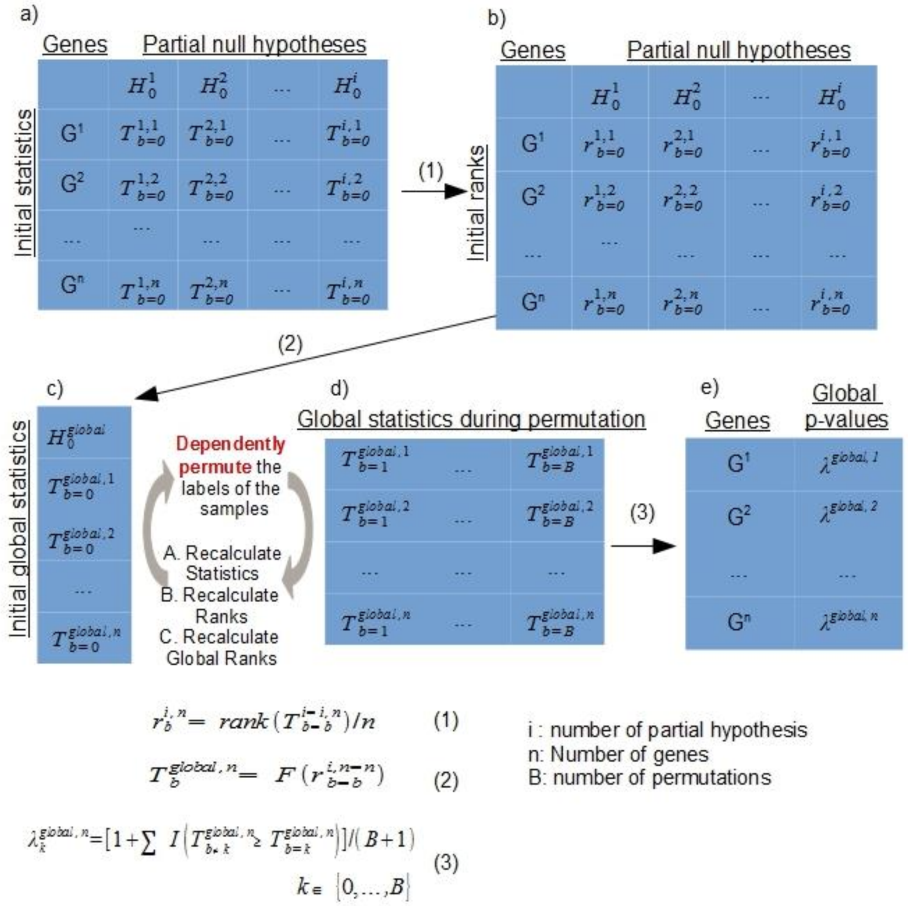


**Fig A: Employing ranks in omicsNPC framework.** a) Divide the question of interest, global null hypothesis, to a set of i partial null hypotheses. For each partial null hypothesis, select a test statistic sensitive to the alternative and for each gene n calculate its value in the observed data, $T_{b=0}^{i,n}$ . Each column is filled with i's partial hypothesis statistics. b) For each column the statistics are ranked in the range of 0 to 1 from the least to the more extreme. c) For each gene, the ranks that belong to the same row are combined through a function F(), like the sum or the product, to calculate the global statistic on the observed data, $T_{b=0}^{globaln}$ . d) The empirical distribution of the global statistic is generated through permutation. Steps A to C are repeated B times by dependently permuting the labels of the samples. e) Finally, for each gene a global p-value is calculated by employing function (3), which compares the value of the global statistic on the observed data against its empirical distribution.

a)

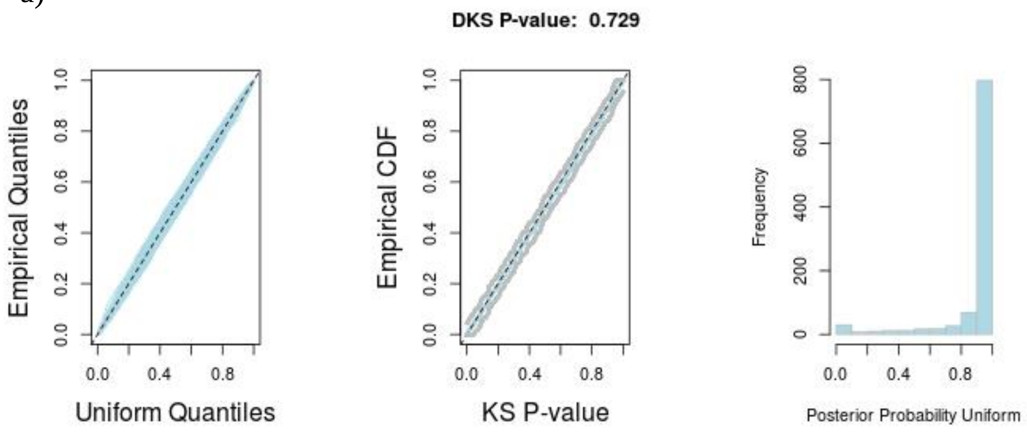


omicsNPC - Fisher

b)

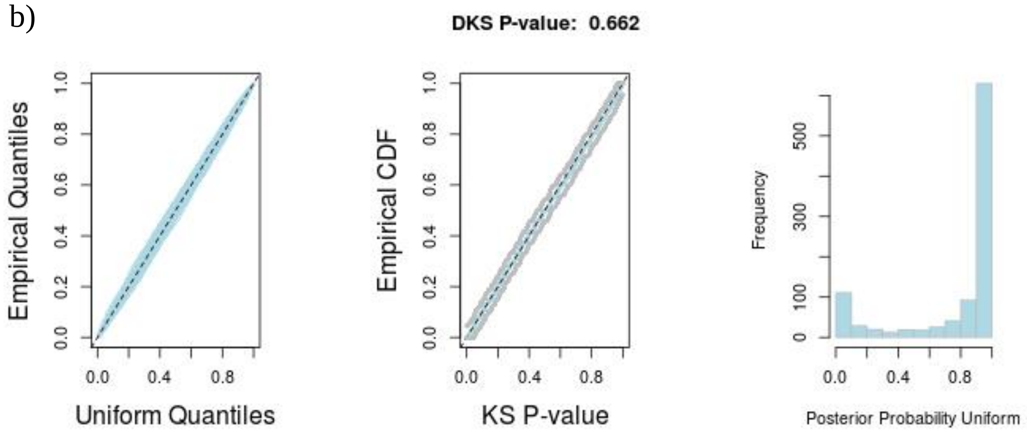


omicsNPC - Liptak

c)

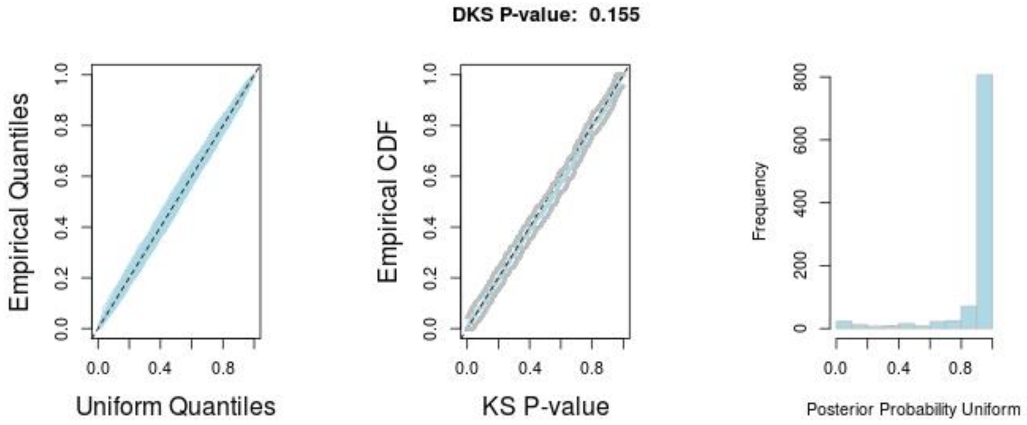


omicsNPC - Tippett

**Fig B: JNC's diagnostics plots on uncorrelated samples employing omicsNPC.** Two uncorrelated microarray datasets were simulated and analysed using omicsNPC. a) Fisher combining function was employed. The diagnostic plots are, from left to right: (i) a quantile-quantile (Q-Q) plot of the p-values for each study versus the uniform quantiles. The lines should be close to the $45^o$ diagonal; lines below the diagonal indicate optimist results, lines above the diagonal conservatives ones; (ii) the empirical distribution function of the first level KS test p-values (blue) with confidence bands (grey), again the line should fall along the $45^o$ line; and (iii) a histogram of the posterior probabilities that each set of p-values is uniform, these values should be near one. b) Liptak combining function was employed. The order of diagnostic plots is the same as a). c) Tippett combining function was employed. The order of diagnostic plots is the same as a). In all cases omicsNPC produced calibrated p-values. Q-Q plots' lines fall on the $45^o$ line, the empirical distribution function of the first level KS test fallen also on the $45^o$ line, the histogram of the posterior probabilities was near one and the dks p-values were not significant, as expected.

a)

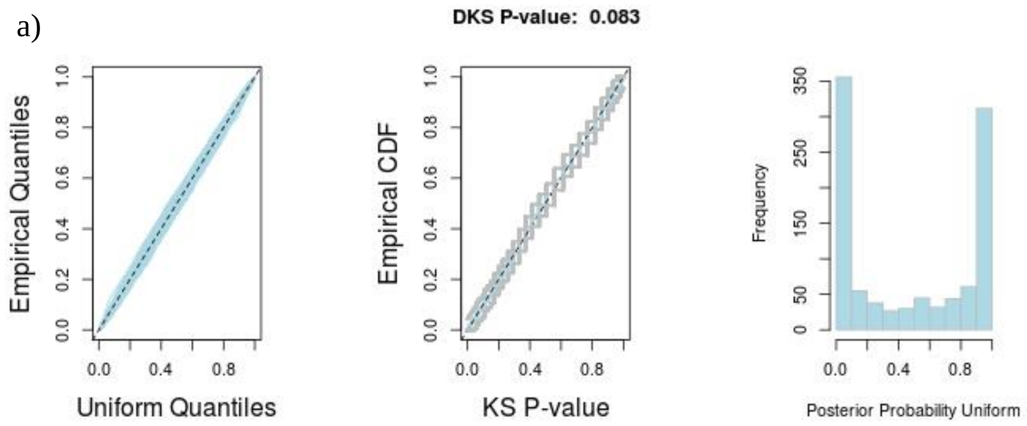


NPC$^{no\text{-}correction}$ - Fisher

b)

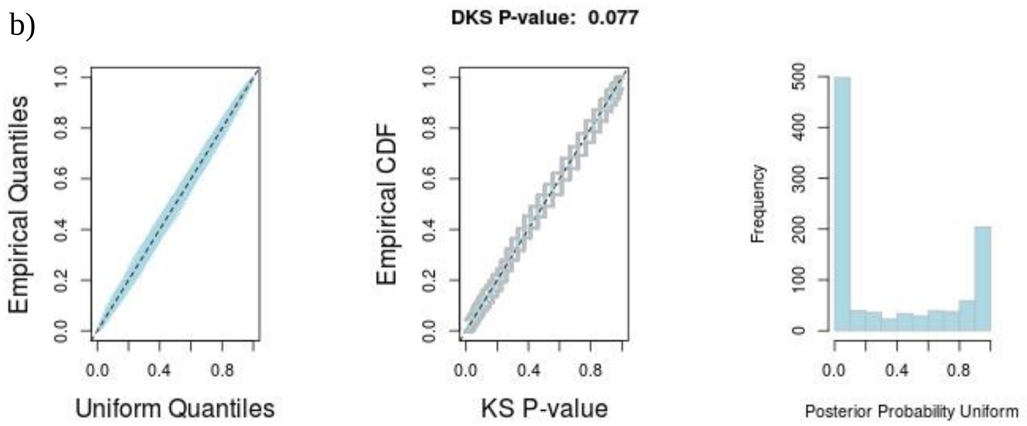


NPC$^{no\text{-}correction}$ - Liptak

c)

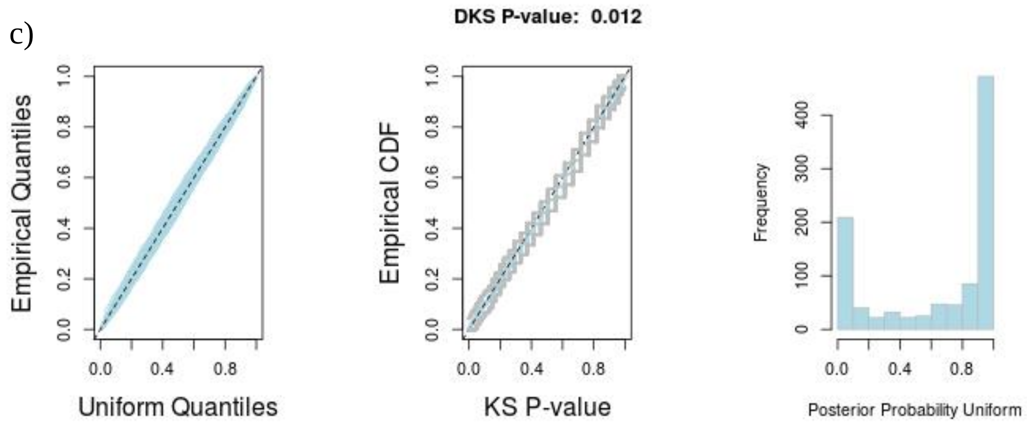


NPC$^{no\text{-}correction}$ - Tippett

**Fig C: JNC's diagnostics plots on uncorrelated samples with NPC$^{no\text{-}correction}$.** Details as in Fig B. In all cases NPC$^{no\text{-}correction}$ produced slightly un-calibrated p-values. Although Q-Q plots' lines fell on the 45$^o$ line and the empirical distribution function of the first level KS test fell also on the 45$^o$ line, the histogram of the

posterior probabilities was greatly deviated between zero and one and the dks p-values were one order of magnitude less than omicsNPC dks p-values. Last, Tippett dks p-value was statistical significant, 0.012.

a)

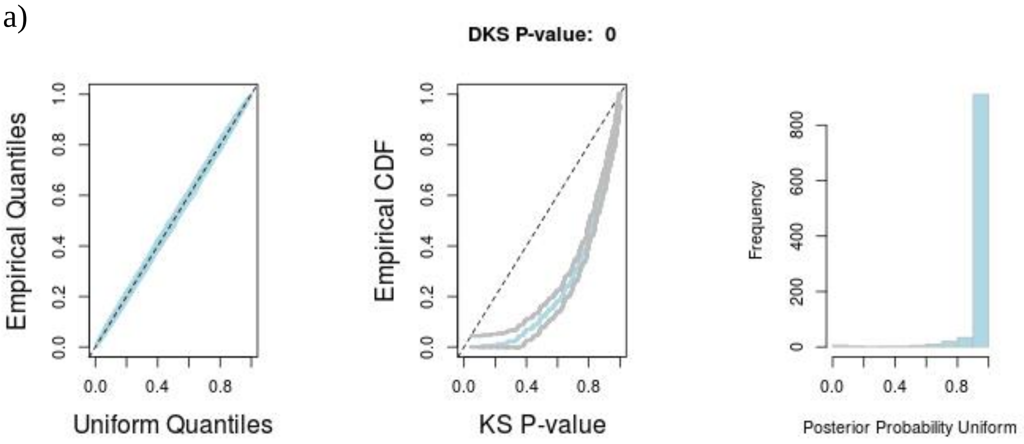


omicsNPC - RankSum

b)

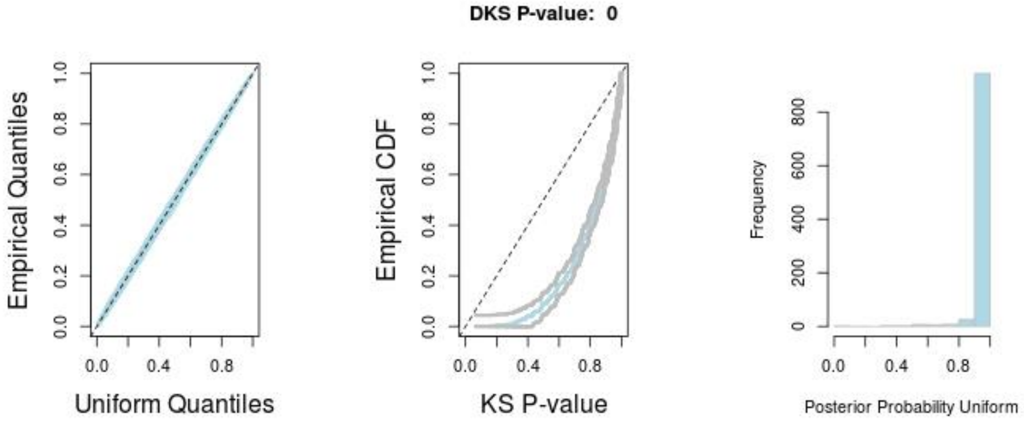


omicsNPC - RankProd

c)

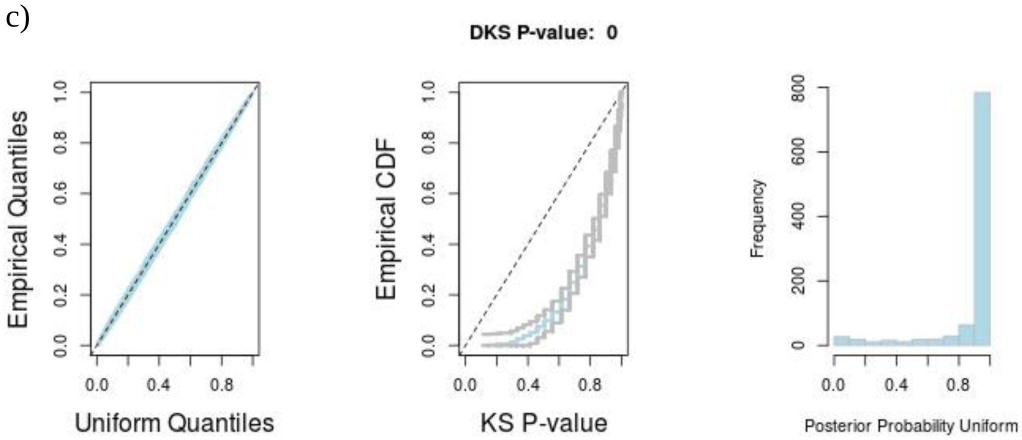


RankProd

**Fig D: JNC's diagnostics plots on uncorrelated samples employing omicsNPC$^{RankSum}$, omicsNPC$^{RankProd}$ , RankProd.** Details as in Fig B. In all cases highly significant dks test p-values are produced.

a)

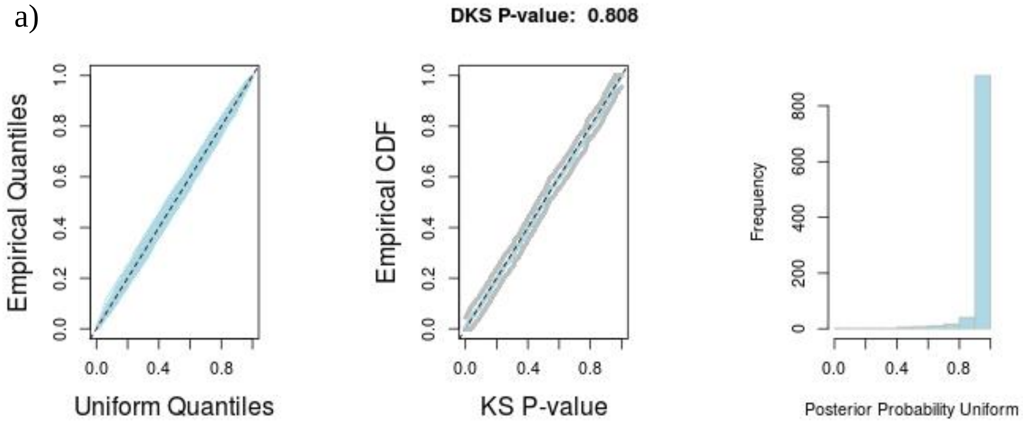


CP – Combining P-values

**Fig E: JNC's diagnostics plots on uncorrelated samples employing combining p-values (CP) approach.** Details as in Fig B, analysed using CP. Combining p-values parametrically produced calibrated p-values on uncorrelated data. Q-Q plots' lines fall on the $45^{o}$ line, the empirical distribution function of the first level KS test fallen also on the $45^{o}$ line, the histogram of the posterior probabilities was near one and the dks p-values were not significant, as expected.

a)

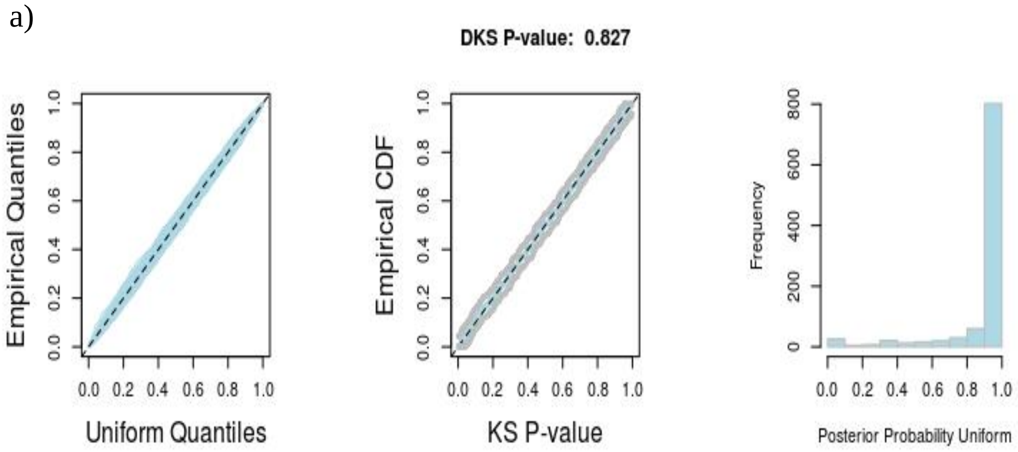


Benjamini

**Fig F: JNC's diagnostics plots on uncorrelated samples employing Benjamini approach.** Details as in Fig B. Benjamini produced calibrated p-values on uncorrelated samples: Q-Q plots' lines fall on the 45$^o$ line, the empirical distribution function of the first level KS test fallen also on the 45$^o$ line, the histogram of the posterior probabilities was near one and the dks p-values were not significant, as expected.

a)

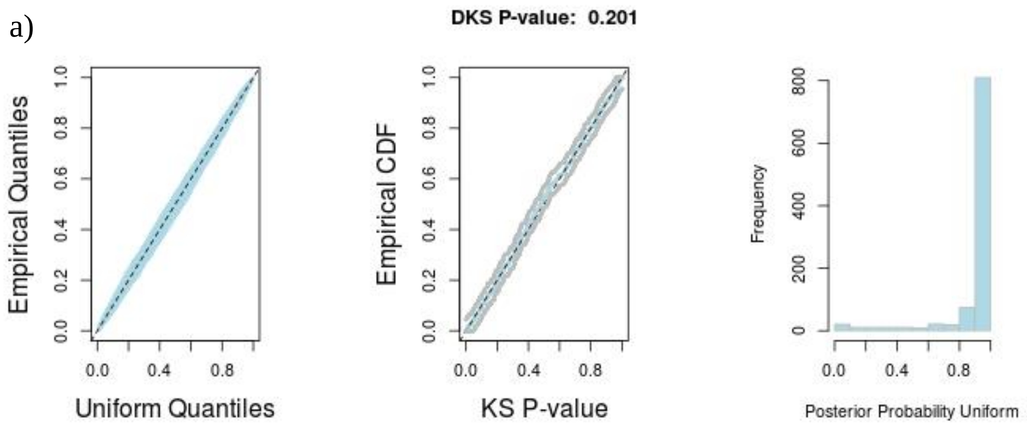


omicsNPC - Fisher

b)

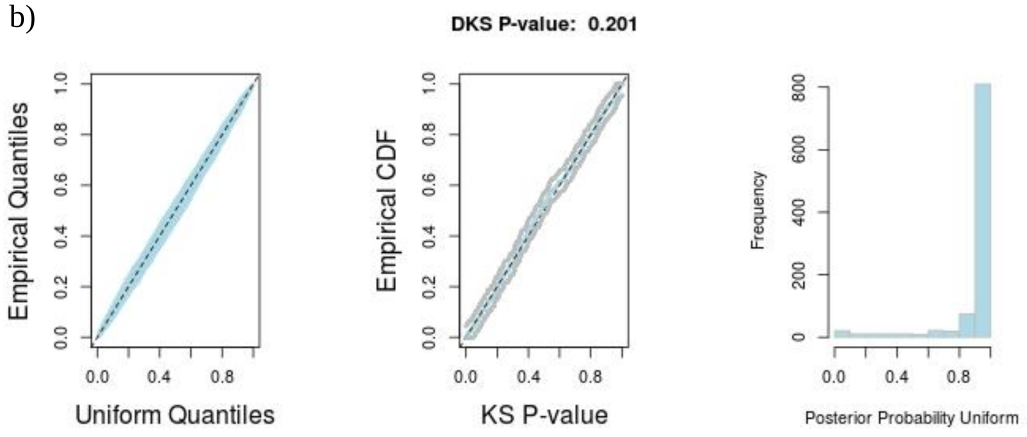


omicsNPC - Liptak

c)

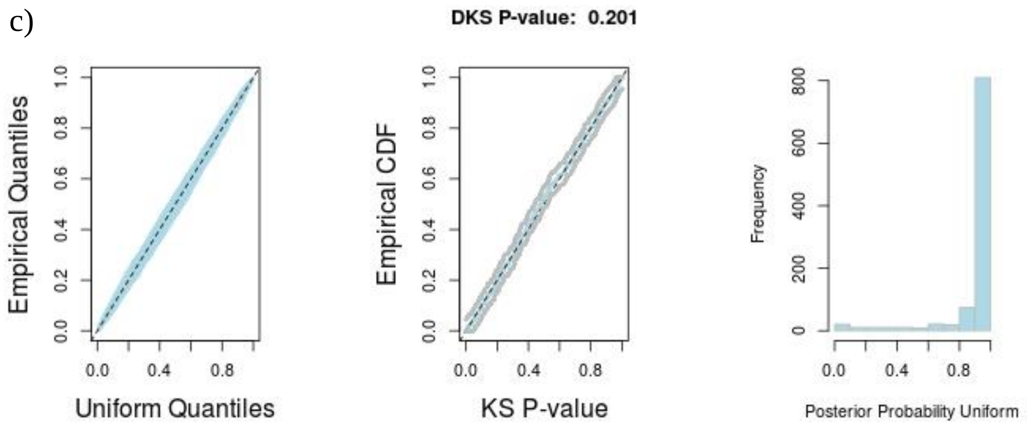


omicsNPC - Tippett

**Fig G: JNC's diagnostics plots on correlated samples employing omicsNPC.** Two perfectly correlated microarray datasets were simulated and analysed using omicsNPC. a) Fisher combining function was employed. The diagnostic plots are, from left to right: (i) a quantile-quantile (Q-Q) plot of the p-values for each study

versus the uniform quantiles, these lines should be close to the 45$^o$ diagonal; lines below the diagonal indicate optimist results, lines above the diagonal conservatives ones; (ii) the empirical distribution function of the first level KS test p-values (blue) with confidence bands (grey), again the line should fall along the 45$^o$ line; (iii) a histogram of the posterior probabilities that each set of p-values is uniform these values should be near one. b) Liptak combining function. The order of diagnostic plots is the same as a). c) Tippett combining function. The order of diagnostic plots is the same as a). In all cases omicsNPC produced calibrated p-values. Q-Q plots' lines fall on the 45$^o$ line, the empirical distribution function of the first level KS test fallen also on the 45$^o$ line, the histogram of the posterior probabilities was near one and the dks p-values were not significant, as expected.

a)

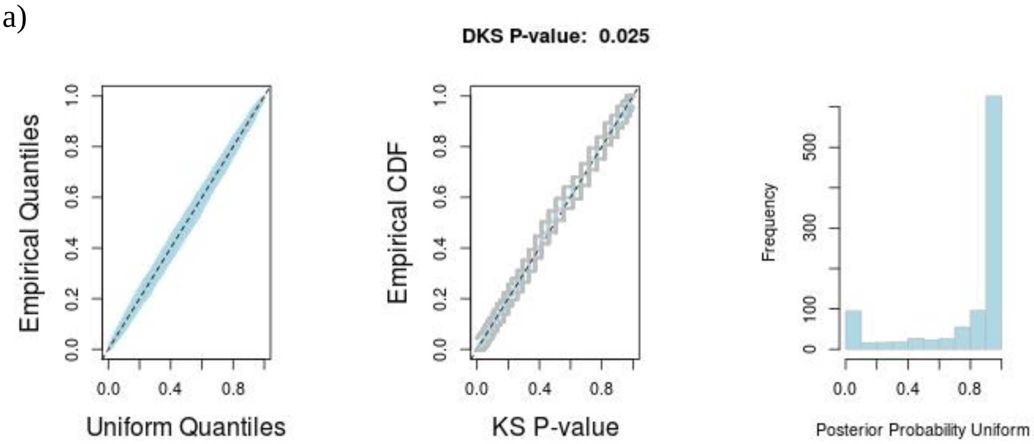


NPC$^{no\text{-}correction}$ - Fisher

b)

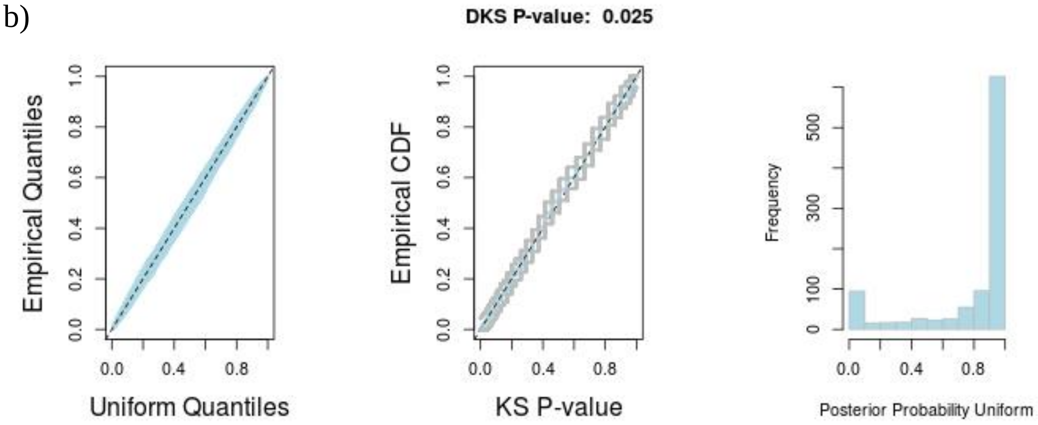


NPC$^{no\text{-}correction}$ - Liptak

c)

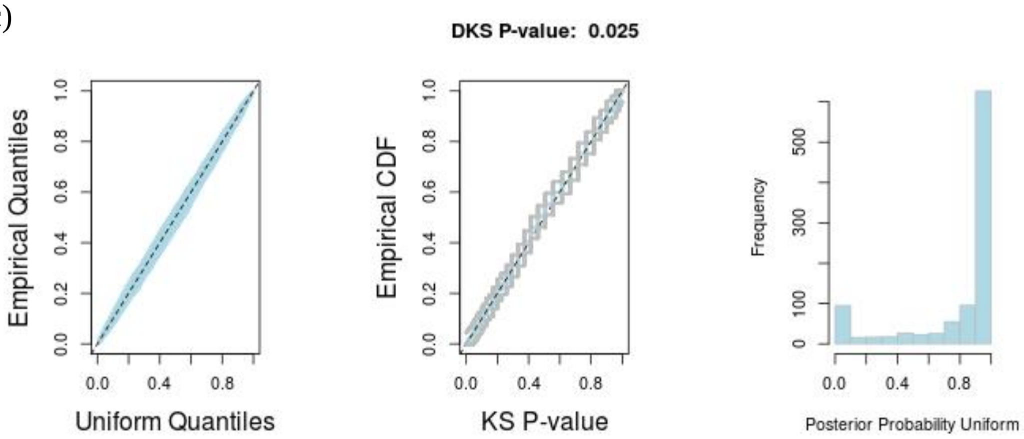


NPC$^{no\text{-}correction}$ - Tippett

**Fig H: JNC's diagnostics plots on correlated samples employing NPC$^{no\text{-}correction}$.** Details as in Fig G. In all cases NPC produced slightly un-calibrated p-values. Although Q-Q plots' lines fall on the 45$^{o}$ line and the empirical distribution function of the first level KS test fallen also on the 45$^{o}$ line, the histogram of the posterior probabilities was sightly deviated between zero and one. Last, all dks p-values were statistical significant and one order of magnitude less than omicsNPC dks p-values.

a)

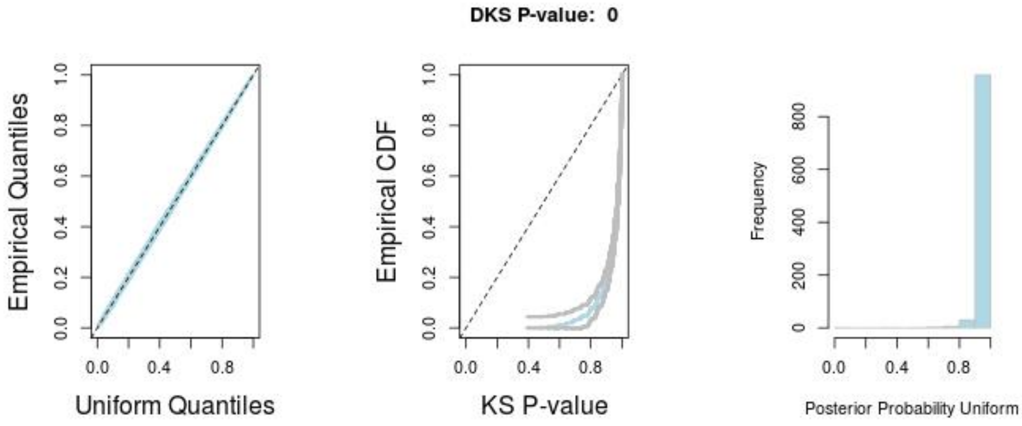


omicsNPC - RankSum

b)

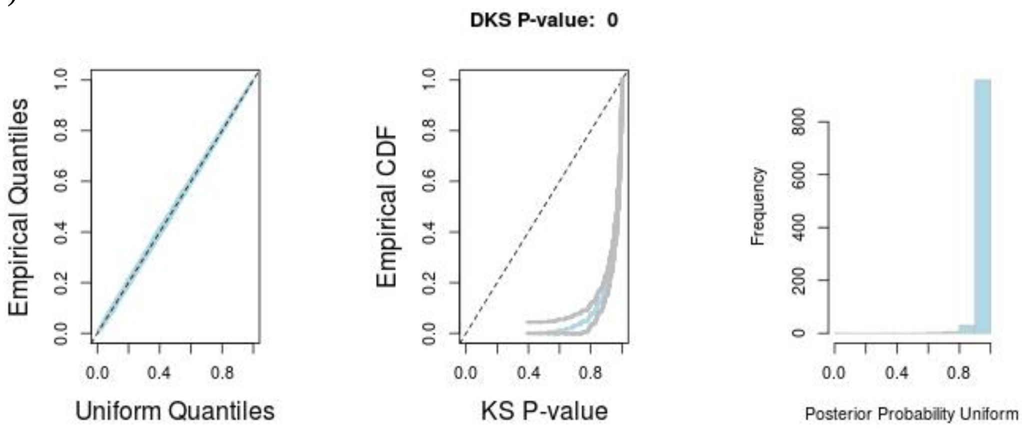


omicsNPC - RankProd

c)

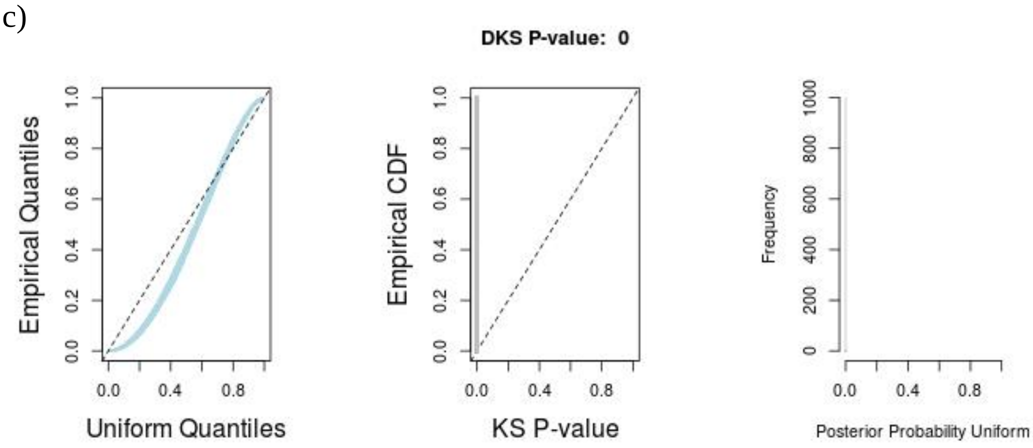


RankProd

**Fig I: JNC's diagnostics plots on correlated samples employing rank approaches.** Details as in Fig G. In all cases the dks p-value is highly significant.

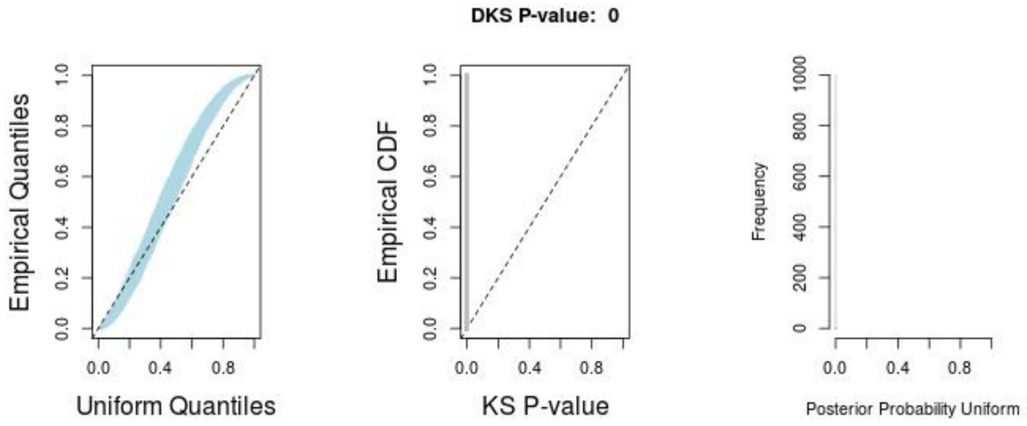


CP – Combining P-values

**Fig J: JNC's diagnostics plots on correlated samples employing combining p-values (CP) approaches.** Details as in Fig G. The dks p-value is highly significant, and the KS p-values are clearly shifted towards zero.

a)

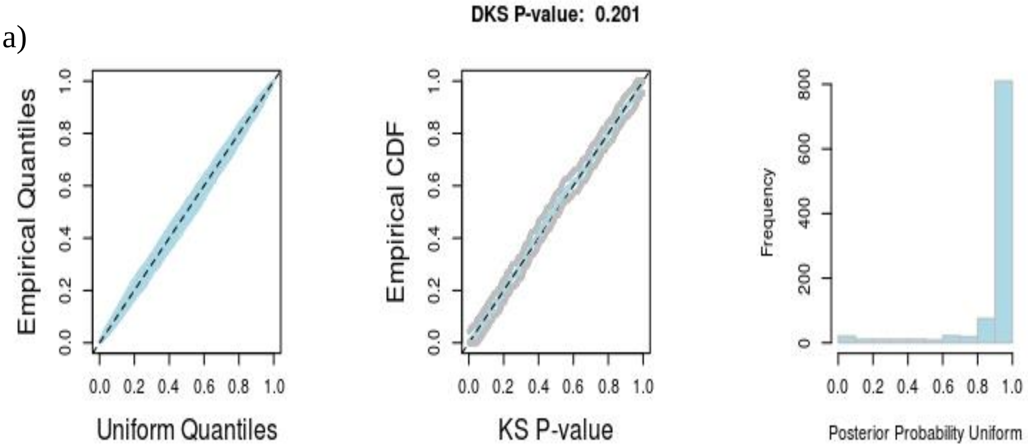


Benjamini

**Fig K: JNC's diagnostics plots on correlated samples employing Benjamini approach.** Details as in Fig F. The Benjamini method produced calibrated p-values: Q-Q plots' lines fall on the 45º line, the empirical distribution function of the first level KS test fallen also on the 45º line, the histogram of the posterior probabilities was near one and the dks p-values were not significant, as expected.

A) B)

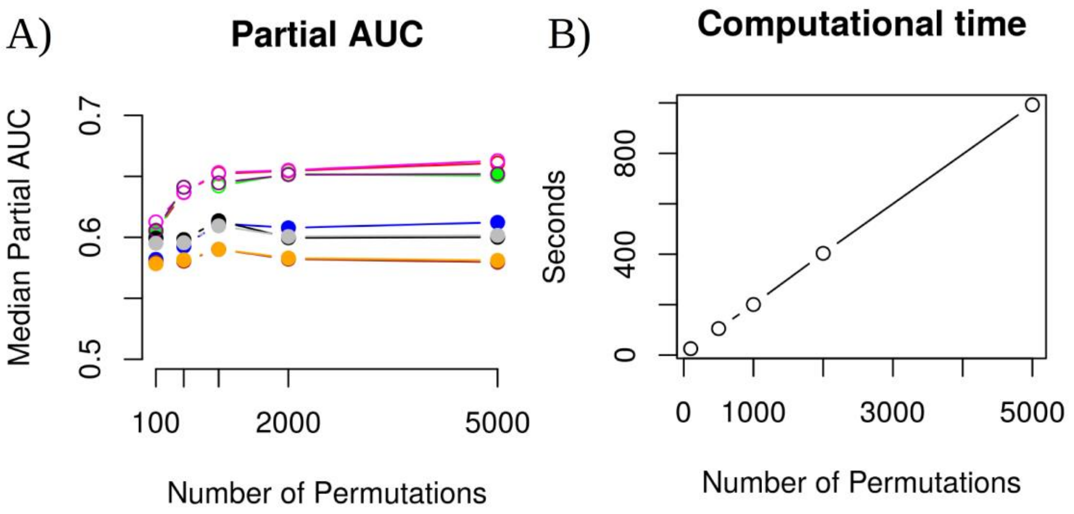


**Fig L: Performance and computational time in relation to the number of permutations.** A) The figure illustrates the median pAUCs of each algorithm in the Different Modalities scenario, when all genes were taken into account. Each line corresponds to a specific method, which analysed the data in combination. Specifically, red – omicsNPC$^{Fisher}$, green – omicsNPC$^{Tippett}$ , blue – omicsNPC$^{Liptak}$, dark magenta – Benjamini, black omicsNPC$^{RankSum}$, brown – omicsNPC$^{RankProd}$ , orange – RankProd, magenta – CP, grey – RankSum_scores. The x axis represents the number of permutations and the y axis the median pAUCs. Filled circles suggest that the observed differences from the best method were statistical significant at level 0.05. B) The figure illustrates the computational time of omicsNPC in relation with the number of permutations. As evident the computational time has a linear relation to the number of permutations.

**Table A: median partial AUCs and significance for increasing sample size and genes (not) deregulated over all data modalities (Different Modalities scenario).**

| | pAUC, s= 4 | Significance | pAUC, s= 6 | Significance | pAUC, s= 8 | Significance | pAUC, s=10 | Significance |
|---|---|---|---|---|---|---|---|---|
| voom+limma | 0.614 | 0 | 0.687 | 0 | 0.621 | 0 | 0.638 | 0 |
| Limma | 0.572 | 0 | 0.592 | 0 | 0.611 | 0 | 0.598 | 0 |
| Chi-square test | 0.597 | 0 | 0.556 | 0 | 0.576 | 0 | 0.551 | 0 |
| Wilcoxon rank sum | 0.558 | 0 | 0.604 | 0 | 0.587 | 0 | 0.555 | 0 |
| OmicsNPC-Fisher | 0.668 | 0 | 0.756 | 0.114 | 0.715 | 0.081 | 0.7 | 0.277 |
| OmicsNPC-Liptak | 0.66 | 0 | 0.702 | 0 | 0.69 | 0 | 0.673 | 0 |
| OmicsNPC-Tippett | 0.608 | 0 | 0.705 | 0 | 0.647 | 0 | 0.666 | 0 |
| OmicsNPC-RankSum | 0.707 | 0.052 | 0.73 | 0 | 0.707 | 0.005 | 0.678 | 0 |
| OmicsNPC-RankProd | 0.673 | 0 | 0.691 | 0 | 0.675 | 0 | 0.647 | 0 |
| RankProd | 0.679 | 0 | 0.692 | 0 | 0.676 | 0 | 0.646 | 0 |
| CP | **0.723** | **1** | **0.77** | **1** | **0.724** | **1** | **0.71** | **1** |
| Benjamini, u=1 | 0.627 | 0 | 0.714 | 0 | 0.652 | 0 | 0.666 | 0 |
| RankSum Scores | 0.708 | 0.174 | 0.735 | 0 | 0.709 | 0.008 | 0.678 | 0 |

Table legend: the table illustrates the median pAUCs of each algorithm in the Different Modalities scenario, on genes that have the same behavior across all modalities, i.e., they are either deregulated in all datasets or in none of them. Each row corresponds to a specific method. The first four rows report the results for each data modality analysed in isolation, whereas the remaining rows report the results obtained from the integrative analysis methods. Labels "pAUCs, s=X" represents the median values for X samples. Significance columns show the p-values from the Wilcoxon two sided tests between each method and the best one. The best performing method for each configuration is reported in bold.

**Table B: median partial AUCs and significance for increasing sample size and all genes (Different Modalities scenario).**

| | pAUC, s= 4 | Significance | pAUC, s= 6 | Significance | pAUC, s= 8 | Significance | pAUC, s= 10 | Significance |
|---|---|---|---|---|---|---|---|---|
| OmicsNPC-Fisher | 0.614 | 0 | 0.68 | 0.461 | 0.656 | 0.499 | 0.647 | 0.583 |
| OmicsNPC-Liptak | 0.59 | 0 | 0.62 | 0 | 0.607 | 0 | 0.603 | 0 |
| OmicsNPC-Tippett | 0.592 | 0 | 0.682 | 0.242 | 0.639 | 0 | 0.651 | 1 |
| OmicsNPC-RankSum | 0.617 | 0 | 0.631 | 0 | 0.612 | 0 | 0.603 | 0 |
| OmicsNPC-RankProd | 0.594 | 0 | 0.61 | 0 | 0.593 | 0 | 0.584 | 0 |
| RankProd | 0.599 | 0 | 0.611 | 0 | 0.593 | 0 | 0.585 | 0 |
| CP | **0.638** | **1** | **0.688** | **1** | **0.657** | **1** | 0.65 | 0.883 |
| Benjamini, u=1 | 0.6 | 0 | 0.684 | 0.429 | 0.642 | 0 | **0.652** | **1** |
| RankSum Scores | 0.618 | 0 | 0.633 | 0 | 0.614 | 0 | 0.601 | 0 |

Table legend: the table illustrates the median pAUCs of each algorithm in the Different Modalities scenario, when all genes were taken into account. Each row corresponds to a specific method. The column "pAUCs, s=X" represents the median values, X equals the number of samples. Significance columns show the p-values from the Wilcoxon two sided tests between each method and the best one. The best performing method for each configuration is reported in bold.

**Table C: median partial AUCs and significance for different number of modalities and genes (not) deregulated over all data modalities (Different Modalities scenario)**.

| | pAUC, m = 2 | Significance | pAUC, m = 3 | Significance | pAUC, m = 4 | Significance |
|---|---|---|---|---|---|---|
| OmicsNPC-Fisher | 0.663 | 0.277 | 0.675 | 0.659 | 0.696 | 0.355 |
| OmicsNPC-Liptak | 0.651 | 0.001 | 0.638 | 0 | 0.663 | 0 |
| OmicsNPC-Tippett | 0.659 | 0.017 | 0.645 | 0 | 0.651 | 0 |
| OmicsNPC-RankSum | 0.656 | 0.017 | 0.638 | 0 | 0.676 | 0 |
| OmicsNPC-RankProd | 0.644 | 0 | 0.619 | 0 | 0.646 | 0 |
| RankProd | 0.644 | 0 | 0.621 | 0 | 0.646 | 0 |
| CP | **0.668** | **1** | **0.681** | **1** | **0.699** | **1** |
| Benjamini, u=1 | 0.659 | 0.018 | 0.646 | 0.001 | 0.656 | 0 |
| RankSum Scores | 0.651 | 0.001 | 0.638 | 0 | 0.672 | 0 |

Table legend: the table illustrates the median pAUCs of each algorithm in the Different Modalities scenario, on genes that have the same behavior across all modalities, i.e., they are either deregulated in all datasets or in none of them. Each row corresponds to a specific method. The column “pAUCs, m = X” represents the median values, X equals the number of modalities used in the analysis. Significance columns show the p-values from the Wilcoxon two sided tests between each method and the best one. The best performing method for each configuration is reported in bold.

**Table D: median partial AUCs and significance for different number of modalities and all genes (Different Modalities scenario).**

| | pAUC, m = 2 | Significance | pAUC, m = 3 | Significance | pAUC, m = 4 | Significance |
|---|---|---|---|---|---|---|
| OmicsNPC-Fisher | 0.654 | 0.547 | 0.654 | 0.841 | 0.645 | 1 |
| OmicsNPC-Liptak | 0.63 | 0 | 0.61 | 0 | 0.599 | 0 |
| OmicsNPC-Tippett | 0.652 | 0.149 | 0.645 | 0.211 | 0.644 | 0.841 |
| OmicsNPC-RankSum | 0.634 | 0 | 0.609 | 0 | 0.604 | 0 |
| OmicsNPC-RankProd | 0.624 | 0 | 0.595 | 0 | 0.585 | 0 |
| RankProd | 0.625 | 0 | 0.597 | 0 | 0.585 | 0 |
| CP | **0.654** | **1** | **0.656** | **1** | 0.643 | 0.989 |
| Benjamini, u=1 | 0.652 | 0.183 | 0.646 | 0.231 | **0.646** | **1** |
| RankSum Scores | 0.627 | 0 | 0.608 | 0 | 0.602 | 0 |

Table legend: the table illustrates the median pAUCs of each algorithm in the Different Modalities scenario, when all genes were taken into account. Each row corresponds to a specific method. The column “pAUCs, m = X” represents the median values, X equals the number of modalities used in the analysis. Significance columns show the p-values from the Wilcoxon two sided tests between each method and the best one. The best performing method for each configuration is reported in bold.

**Table E: median pAUCs and significance for increasing number of permutations (Different Modalities scenario).**

| | pAUC perm= 100 | Significance | pAUC, perm = 500 | Significance | pAUC, perm = 1000 | Significance | pAUC, perm= 2000 | Significance | pAUC, perm= 5000 | Significance |
|---|---|---|---|---|---|---|---|---|---|---|
| OmicsNPC-Fisher | 0.605 | 0.063 | 0.637 | 0.698 | 0.652 | 0.659 | 0.654 | 0.779 | 0.661 | 0.883 |
| OmicsNPC-Liptak | 0.582 | 0 | 0.593 | 0 | 0.611 | 0 | 0.608 | 0 | 0.612 | 0 |
| OmicsNPC-Tippett | 0.604 | 0.038 | **0.641** | **1** | 0.642 | 0.134 | 0.652 | 0.231 | 0.651 | 0.049 |
| OmicsNPC-RankSum | 0.599 | 0.001 | 0.598 | 0 | 0.614 | 0 | 0.6 | 0 | 0.6 | 0 |
| OmicsNPC-RankProd | 0.579 | 0 | 0.58 | 0 | 0.59 | 0 | 0.582 | 0 | 0.58 | 0 |
| RankProd | 0.578 | 0 | 0.582 | 0 | 0.59 | 0 | 0.583 | 0 | 0.581 | 0 |
| CP | **0.613** | **1** | 0.637 | 1 | **0.653** | **1** | **0.655** | **1** | **0.663** | **1** |
| Benjamini, u=1 | 0.606 | 0.121 | 0.641 | 0.925 | 0.645 | 0.201 | 0.651 | 0.301 | 0.652 | 0.076 |
| RankSum Scores | 0.595 | 0 | 0.596 | 0 | 0.609 | 0 | 0.601 | 0 | 0.602 | 0 |

Table legend: the table illustrates the median pAUCs of each algorithm in the Different Modalities scenario, when all genes were taken into account. Each row corresponds to a specific method. The column "pAUCs, perm = X" represents the median values, X equals the number of permutations used in the analysis. Significance columns show the p-values from the Wilcoxon two sided tests between each method and the best one. The best performing method for each configuration is reported in bold.

**Table F: median pAUCs and significance (Correlated Modalities scenario).**

| | pAUC, cor=0.6 | Signifi-cance | pAUC, cor=0.7 | Signifi-cance | pAUC, cor=0.8 | Signifi-cance | pAUC, cor=0.9 | Signifi-cance | pAUC, cor=1 | Signifi-cance |
|---|---|---|---|---|---|---|---|---|---|---|
| Single dataset 1 – Limma | 0.595 | 0 | 0.596 | 0 | 0.592 | 0 | 0.595 | 0 | 0.597 | 0 |
| Single dataset 2 – Limma | 0.593 | 0 | 0.596 | 0 | 0.601 | 0 | 0.604 | 0 | 0.598 | 0 |
| OmicsNPC-Fisher | **0.647** | **1** | 0.648 | 0.678 | 0.647 | 0.968 | **0.649** | **1** | **0.651** | **1** |
| OmicsNPC-Liptak | 0.646 | 1 | **0.649** | **1** | **0.648** | **1** | 0.649 | 0.779 | 0.651 | 0.602 |
| OmicsNPC-Tippett | 0.619 | 0 | 0.622 | 0 | 0.62 | 0 | 0.626 | 0 | 0.623 | 0 |
| OmicsNPC-RankSum | 0.62 | 0 | 0.627 | 0 | 0.625 | 0 | 0.631 | 0 | 0.633 | 0 |
| OmicsNPC-RankProd | 0.618 | 0 | 0.626 | 0 | 0.622 | 0 | 0.631 | 0 | 0.632 | 0 |
| RankProd | 0.584 | 0 | 0.576 | 0 | 0.558 | 0 | 0.548 | 0 | 0.534 | 0 |
| CP | 0.614 | 0 | 0.605 | 0 | 0.593 | 0 | 0.586 | 0 | 0.579 | 0 |
| Benjamini, u=1 | 0.625 | 0 | 0.633 | 0 | 0.633 | 0.015 | 0.645 | 0.094 | 0.627 | 0 |
| RankSum Scores | 0.587 | 0 | 0.576 | 0 | 0.558 | 0 | 0.55 | 0 | 0.537 | 0 |

Table legend: the table illustrates the median pAUCs of each algorithm in the correlated modalities scenario. Each row corresponds to a specific method. The column “pAUCs, cor = X” represents the median values, X represents the level of correlation introduced in the data. Significance columns show the p-values from the Wilcoxon two sided tests between each method and the best one. The best performing method for each configuration is reported in bold.

**Table G. Differentially Expressed Genes in the BRCA data.**

| FDR | RNAseq | RNAseqV2 | Exp-Gene | Fisher | Liptak | Tippett |
|---|---|---|---|---|---|---|
| 0.01 | 5205 | 5178 | 3756 | 5523 | 5284 | 5823 |
| 0.03 | 6437 | 6406 | 4936 | 6890 | 6633 | 7195 |
| 0.05 | 7116 | 7091 | 5674 | 7721 | 7429 | 8027 |
| 0.07 | 7594 | 7596 | 6236 | 8312 | 8012 | 8603 |
| 0.1 | 8153 | 8155 | 6851 | 8982 | 8656 | 9270 |
| 0.11 | 8331 | 8332 | 7048 | 9192 | 8857 | 9448 |

Table legend: results according to different single-dataset analyses or omicsNPC with different combining functions (columns) and various FDR thresholds in the range [0.1 – 0.11] (rows). Combining data types with omicsNPC leads to a higher number of finding, independently by the threshold and combining function used.
